# Comparative preclinical drug response analyses of T-prolymphocytic leukemia reveal no differences between known gene expression subgroups

**DOI:** 10.1186/s13062-025-00701-3

**Published:** 2025-10-27

**Authors:** Nathan Mikhaylenko, Till Braun, Sanna Timonen, Satu Mustjoki, Marco Herling, Michael Seifert

**Affiliations:** 1 Institute for Medical Informatics and Biometry (IMB), Faculty of Medicine Carl Gustav Carus, Technische Universität Dresden, Fetscherstr. 74, D-01307 Dresden, Germany; 2 Department I of Internal Medicine, Center for Integrated Oncology Aachen-Bonn-Cologne-Duesseldorf, University Hospital Cologne, University of Cologne, Kerpener Str. 62, D-50937 Cologne, Germany; 3 Hematology Research Unit Helsinki, University of Helsinki and Helsinki University Hospital Comprehensive Cancer Center, Haartmaninkatu 8, Helsinki, FIN-00290 Finland; 4 Department of Hematology, Cellular Therapy, Hemostaseology, Infectious Diseases, University Hospital Leipzig, University of Leipzig, Liebigstr. 27, D-04103 Leipzig, Germany

**Keywords:** T-prolymphocytic leukemia (T-PLL), Computational drug response analysis, Gene expression subgroups

## Abstract

**Background:**

T-prolymphocytic leukemia (T-PLL) is a rare mature T-cell neoplasm with poor prognosis that mainly affects elderly people. Alemtuzumab is widely considered as first-line therapy, but almost all patients relapse within one year, if not consolidated by an allogeneic stem cell transplantation. The improved understanding of T-PLL-specific molecular pathomechanisms gained over the last years suggested new potential therapeutic targets (epigenetic dysregulation, defective DNA damage response, aberrant cell cycle regulation, dysregulated prosurvival pathways), which were recently evaluated in a preclinical drug response study. In addition, existing genomewide T-PLL gene expression profiles enabled the identification of three robust T-PLL gene expression subgroups differing in cellular processes like immune response, cellular respiration, cell proliferation, apoptosis, or migration. So far, these T-PLL gene expression subgroups were not considered in preclinical drug response analyses, but recently published drug response screens and corresponding already publicly available gene expression profiles offer the great opportunity to integrate both data types. Therefore, we computationally analyzed samples from 34 T-PLL patients of two comparable cohorts for their response to in total 11 drugs.

**Results:**

No T-PLL subgroup-specific differences or sex differences in response to the tested drugs were found. With respect to the underlying drug dosage schemes, venetoclax and cladribine were most effective in erasing T-PLL cancer cells among both cohorts. Also dinaciclib, idasanutlin, romidepsin, and KRT-232, which were only tested in one of both cohorts, were very effective for all or most of the T-PLL patient samples. For the three drugs bendamustine, cladribine, and fludarabine, which were only effective in a subset of the T-PLL samples, an exploratory differential gene expression analysis predicted drug-specific genes that distinguished between strongly and not strongly responding samples. In-depth annotation and literature analyses showed that many of these genes are known to play a role in leukemias or other types of cancer. Many of these genes were also confirmed by a direct correlation analysis between gene expression levels and drug responses.

**Conclusions:**

The absence of T-PLL gene expression subgroup-specific drug responses across the tested drugs can be important for the design of future drug screens and may ease potential clinical trials. Genes associated with drug-specific responses could be useful for improved patient stratification and may help to characterize molecular settings associated with effective responses.

**Clinical trial number:**

Not applicable.

**Supplementary Information:**

The online version contains supplementary material available at 10.1186/s13062-025-00701-3.

## Background

T-prolymphocytic leukemia (T-PLL) is a mature T-cell leukemia with aggressive clinical course and high mortality rates  [1–5]. With about two cases per one million people per year T-PLL is a rare disease, but T-PLL still represents the most frequent mature T-cell leukemia in Europe and North America  [6, 7]. T-PLL is mainly a disease of elderly people with a median age of 65 years at diagnosis  [4, 8]. The median overall survival of T-PLL patients is less than three years  [3, 9, 10].

T-PLL was first described more than 50 years ago  [11]. Nowadays, uniform T-PLL diagnosis criteria are considered focusing on the histological presence of clonal prolymphocytic T-cells, the presence of complex chromosomal aberrations (e.g. inversions or translocations of chromosome 14), and the typical clinical representation (e.g. exponentially rising lymphocyte counts, splenomegaly, lymphadenopathy) to distinguish T-PLL from other T-cell leukemias  [7, 12–14].

Complex karyotypes triggered by chromosomal inversions, translocations or DNA copy number aberrations are found in T-PLL cancer cells of the majority of patients  [4, 5, 15–18]. A characteristic overexpression of the proto-oncogene *TCL1A* at chromosome 14q32.1 due to an inversion (inv(14)(q11;q32)) or a translocation (t(14;14)(q11;q32))  [4, 5, 17, 19, 20] is frequently found in combination with an inactivation of the tumor suppressor *ATM* at chromosome 11q22.3 by deletions and/or missense mutations  [10, 14, 17]. This joint alteration of *TCL1A* and *ATM* contributes to impaired DNA damage repair and abrogated p53-mediated cell death and therefore most likely represents a major driving force for the initiation of T-PLL development in many patients  [10, 17]. However, the mutational landscape of T-PLL is complex and not fully understood. There are also T-PLL patients that do not show an activation of *TCL1A*  [8, 17]. Other recurrent translocations affecting *MTCP1* (a homolog of *TCL1A*) or haploinsufficiency of *CDKN1B* can also contribute to T-PLL development by influencing cell cycle, apoptosis and DNA repair  [4]. Recurrent mutations of epigenetic regulators (*EZH2*, *TET2*, *BCOR*), of the DNA damage regulator *CHEK2*, of genes of the JAK-STAT signaling pathway (*IL2RG*, *JAK1*, *JAK3*, *STAT5B*), and amplifications of the *MYC* oncogene and *AGO2* involved in RNA interference can also contribute to T-PLL development  [17, 21–24]. Further, heterogeneous expression of oncogenic microRNAs in T-PLL potentially impacting on TGF-beta signaling  [25] and three robust T-PLL gene expression subgroups  [18] were revealed. This whole spectrum of different molecular alterations and disease subgroups clearly emphasizes the complexity of T-PLL, but such an in-depth molecular characterization can also help to develop new targeted therapeutic approaches.

Alemtuzumab, a monoclonal anti-CD52 antibody, is widely considered as first-line therapy for T-PLL patients that induces major responses in about 90% of patients, but almost all patients relapse within one year  [3, 26, 27]. T-PLL relapses are hard to treat, because second-line treatment options are mostly inefficient and limited sensitivity to conventional cytostatics or small molecules are known  [28–32]. The only curative therapy option that currently exists is an allogeneic hematopoietic stem cell transplantation, but only about 30% to 50% of the T-PLL patients are eligible for this therapy due to the increased age of T-PLL patients  [3, 33]. Therefore, strong efforts were made over the last years to identify novel compounds or combinations of compounds to target T-PLL cancer cells  [3, 10, 34–38].

Based on a large T-PLL patient cohort  [17], we were already able to predict three robust T-PLL gene expression subgroups and potential subgroup-specific major regulators known to be involved in the regulation of cellular processes such as immune response, cellular respiration, cell proliferation, apoptosis, or migration  [18]. A part of the T-PLL patients that formed the basis for our gene expression subgroup prediction were also part of a recently published study by von Jan et al. (2024), who performed drug screens to develop optimized preclinical therapeutic concepts for T-PLL  [38]. Such drug screens offer the great opportunity for us to extend our work by analyzing for the first time how our three revealed T-PLL subgroups respond to different drugs.

**Fig. 1:**
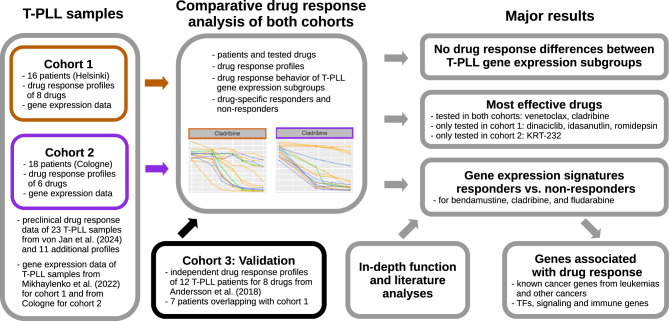
Overview of the performed computational drug response analysis. Drug response profiles and corresponding gene expression profiles of T-PLL samples of in total 34 patients from two cohorts were considered for a comparative drug response analysis. Tested drugs and drug responses profiles of both cohorts were compared. Treatment naive gene expression profiles were used to assign each patient to one of three known T-PLL gene expression subgroups from Mikhaylenko et al. (2022)  [18]. No subgroup-specific differences in drug responses were observed for the tested drugs. The most effective drugs were determined across both cohorts and for each of the two cohorts. For drugs to which only a subset of T-PLL samples responded, differentially expressed genes that distinguished potential responders from non-responders were determined and considered for an in-depth functional and literature analysis. Independently measured drug response profiles of a third cohort from Andersson et al. (2018)  [34] were used to validate the observed drug response behavior of both considered cohorts

Here, we analyze samples from 34 patients of two T-PLL data cohorts for their response to a set of 11 drugs of which three drugs were tested in both cohorts (Fig. 1). We systematically analyze similarities and differences of the drug responses of both cohorts, drug response behavior of the three T-PLL gene expression subgroups, and sex differences in drug response. We further perform exploratory differential gene expression analyses comparing T-PLL samples that strongly respond to a specific drug to those that do not strongly respond coupled with an in-depth literature analysis of response-associated genes. We further complement this by a direct analysis of correlations between gene expression levels and drug response behavior of T-PLL samples. Finally, we compare the drug response behavior of our two main T-PLL cohorts to a third publicly available drug response screen of T-PLL patients from Andersson et al. (2018)  [34]. Our study contributes to a better characterization of the therapeutic potential of specific drugs for T-PLL treatment.

## Methods

### Drug response data and gene expression profiles of T-PLL patients

Drug response data of two T-PLL patient cohorts were provided by the Mustjoki laboratory (first cohort) and by the Herling laboratory (second cohort) to analyze the drug response behavior of the three revealed T-PLL gene expression subgroups from our initial study  [18]. The first cohort comprises data of 16 T-PLL patients from the Mustjoki laboratory in Helsinki (Table 1). These patients were already part of our initial multi-omics T-PLL study that revealed three T-PLL gene expression subgroups  [18]. The drug response profiles of all 16 T-PLL patients were included in  [38]. Peripheral blood mononuclear cells of these 16 patients were cultured in a mononuclear cell medium. The cultured cells were screened for drug response sensitivity considering eight drugs (bendamustine, cladribine, dinaciclib, ibrutinib, idasanutlin, romidepsin, ruxolitinib, venetoclax) utilizing seven doses (Supplementary Fig. S1). Benzethonium chloride (BzCl) was used as positive control (total killing of cells) and dimethyl sulfoxide (DMSO) was used as negative non-effective control. Methodological details to the underlying experimental procedures are reported in  [38]. The drug response data in Supplementary Table S2a quantifies the viability of the cells (percentage of viable cells) at the different drug doses. The processed gene expression profiles obtained by RNA sequencing (RNA-seq) are available in Supplementary Table S5a. Purified T-cells obtained from peripheral blood of T-PLL patients formed the basis of the gene expression data. The second cohort comprises data of 18 T-PLL patients from the Herling laboratory in Cologne (Table 1). The drug response profiles of 7 of the 18 T-PLL patients were already included in  [38]. Again, as described before, cultured peripheral blood mononuclear cells of these 18 patients were screened for drug response sensitivity considering six drugs (belinostat, bendamustine, cladribine, fludarabine, KRT-232, venetoclax) utilizing five doses (Supplementary Fig. S1). The drug response data are provided in Supplementary Table S2b. The gene expression profiles of purified T-cells obtained from peripheral blood of the T-PLL patients are available in Supplementary Table S5b. Corresponding raw RNA-seq data of these samples were provided by the Herling laboratory and processed as described in  [18] utilizing the basic pipeline established in  [39]. A third cohort of T-PLL patients was taken from a related study by Andersson et al. (2018)  [34] to validate the findings of the two other cohorts. This cohort comprises data of 12 T-PLL patients that were tested for their responses to 11 drugs of which eight overlapped with the drugs that were also part of the drug response screens of the first and/or second cohort (Supplementary Fig. S1). Among these 12 patients were seven patients that were part of the first T-PLL cohort. Also after batch correction the available microarray gene expression profiles of the 12 T-PLL patients were not comparable to the gene expression profiles of the two other cohorts. Therefore, only the drug response profiles of this cohort were considered. The drug response data are provided in Supplementary Table S2c.

**Table 1:**
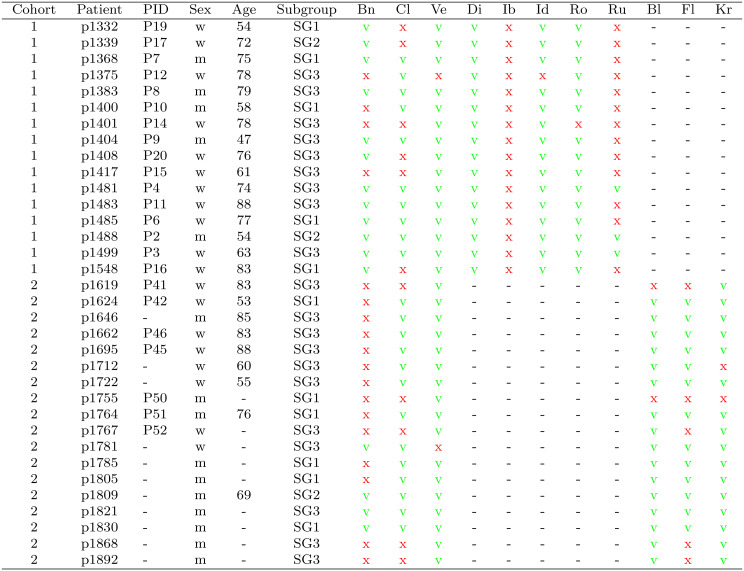
Overview of the two considered T-PLL patient cohorts. PID: corresponding identifiers of patients that were also part of the drug response study by von Jan et al. (2024)  [38]. Subgroup: assignment of patients to one of the three known T-PLL gene expression subgroups from  [18]. Drugs: bn: bendamustine, Cl: cladribine, Ve: venetoclax, Di: dinaciclib, ib: ibrutinib, id: idasanutlin, Ro: romidepsin, Ru: ruxolitinib, bl: belinostat, Fl: fludarabine, Kr: KRT-232. The response of each patient-specific T-PLL sample to a specific drug is marked by a green ‘v’ if the sample strongly responded (cell viability dropped below 50%), by a red ‘x’ if the sample did not strongly respond (cell viability stayed above 50%), and by a black ‘-’ if the sample was not tested for this specific drug

### Assignment of patients to known T-PLL gene expression subgroups

Each T-PLL patient of the first cohort was previously assigned to one of three revealed T-PLL gene expression subgroups based on its cluster membership in the genomewide hierarchical clustering of patient-specific T-PLL gene expression profiles  [18]. Similarly, each T-PLL patient of the second cohort was assigned to its most likely underlying T-PLL gene expression subgroup  [18]. This was done by computing all pairwise Pearson correlation coefficients between the gene expression profile of a patient in the second cohort in relation to each gene expression profile of the 68 original T-PLL patients from  [17], which formed the basis of the hierarchical clustering in  [18]. Prior to this correlation analysis, batch adjustment was done between the original microarray gene expression profiles and the RNA-seq gene expression profiles using the R-package sva  [40]. Finally, each patient of the second cohort was assigned to the T-PLL subgroup of the original patient with the greatest positive correlation coefficient. The subgroup assignments of the T-PLL patients of the first and the second cohort are included in Table 1.

### Drug response analysis of T-PLL patients

The drug response analysis was performed in several steps. First, cancer cell viability curves were visualized by a four-parameter log-logistic model fit computed with the help of the R package drc  [41] to enable comparisons of the obtained drug response profiles between the drugs, the T-PLL gene expression subgroups, and the patient cohorts. For statistical comparisons, the following parameters were obtained from each T-PLL patient sample: the median effective dose ED50 (drug concentration that caused the cell viability to drop down to 50% from the negative control) calculated based on the fitted models using the R package drc  [41], the area under the drug response curve AUC calculated by the R package PharmacoGx  [42], and the drug-specific sensitivity score DSS calculated by the R package Breeze  [43]. Further, for pairwise comparisons between the two cohorts whose T-PLL patient samples were screened at different drug concentrations, the cell viability at the highest common dose was used as comparative score. Alternatively, the area under the drug response curve (AUC) was computed for the longest common dose interval of a drug tested in two or three of the considered cohorts (see Supplementary Fig. S1 for overlapping doses). This overcomes potential limitations of only considering the highest common dose for a comparison of cohorts. The corresponding AUCs were calculated for all T-PLL samples using the auc function of the R package MESS  [44] with natural cubic spline interpolation based on the estimated drug response curves for the common drug dose intervals. The estimated drug response curves represented the initially obtained four-parameter log-logistic model fits obtained based on log_10_-dose inputs and corresponding measured cell viabilities. The obtained AUC values are provided in Supplementary Table S7.

### Differential expression analysis between potential responders and non-responders

For an exploratory comparative gene expression analysis between T-PLL patients whose T-PLL samples strongly responded to a drug treatment and those whose T-PLL samples did not strongly respond, the response behavior of the patient-specific T-PLL samples was used to define potential responders and non-responders. A T-PLL patient was considered as a potential responder to a specific drug if its cancer cell viability dropped below 50% from the negative control baseline during the drug response experiment. A potential non-responder to a specific drug was considered to be a T-PLL patient whose cell viability stayed above 50% for any applied drug dose. This grouping of patients into potential responders and potential non-responders was motivated by the ED50 value, because the ED50 value only exists for patients whose T-PLL samples strongly responded to a specific drug with respect to the underlying dosage scheme. Based on this classification, it was possible to perform an exploratory differential gene expression analysis between potential responders and non-responders for three drugs (bendamustine, cladribine, fludarabine). The differential gene expression analysis between responders and non-responders was done for each T-PLL patient cohort separately or for the joined expression data set of the first and second cohort following limma’s standard workflow  [45]. The results of the differential gene expression analyses are provided in Supplementary Table S3. Please change this and include the 3 that currently stands at the end without being include in the link name."3. Volcano plots were used to visualize the results. Heatmaps (with modified R function heatmap.3) of differentially expressed genes were used to demonstrate the separation capacity between responders and non-responders. Hierarchical clustering within the heatmap was performed using Ward’s linkage criterion (ward.D2)  [46] in combination with one minus the Pearson correlation between two gene expression profiles as basic distance measure.

### Functional enrichment analysis of differentially expressed genes

Basic cancer-relevant gene annotations and pathway annotations were taken from  [18]. The utilized pathway annotations are provided in Supplementary Table S6. Pathways and functional categories were considered to test for an enrichment of differentially expressed genes in each specific annotation category. For each comparison of responders to non-responders, the number of differentially expressed genes in each annotation category was counted separately for down- and up-regulated genes (Supplementary Table S3, $p \le 0.05$). Significance of enrichment per annotation category was determined using Fisher’s exact test (R function fisher.test). Correction for multiple testing was done by computing FDR-adjusted p-values (q-values)  [47] using the R function p.adjust. Bar plots were used to represent the results and to label significantly enriched pathways.

### Correlations between gene expression and drug response

An additional correlation analysis was done to directly associate patient-specific gene expression levels with drug-specific responses to overcome potential limitations of the discrete classification of T-PLL patient samples as strongly or not strongly responding to a specific drug, which was previously considered for the exploratory differential gene expression analysis. Therefore, the Pearson correlation was computed between the expression levels of a gene across all T-PLL patients and their corresponding patient-specific responses to a specific drug (R function cor.test). For bendamustine and cladribine, which were tested in both cohorts, the patient-specific area under the drug response curve for the longest common dose interval of both cohorts was used as drug-specific response measure in the correlation analysis. For fludarabine, which was only tested in the second cohort, the full area under the drug response curve was considered. The obtained gene-specific correlations for each drug, corresponding p-values and FDR-adjusted p-values are provided in Supplementary Table S8.

## Results

### Similarities and differences between patients, drugs, and drug responses of both T-PLL cohorts

Single-agent based drug response profiles of T-PLL samples of two patient cohorts of in total 34 patients were considered for an in-depth comparative analysis of both cohorts (Fig. 1). An overview of the T-PLL patients of the two cohorts is provided in Table 1. The first cohort comprised 16 T-PLL patients and the second cohort contained 18 patients. The included drugs were specifically selected in  [38] to target new actionable T-PLL vulnerabilities (epigenetic dysregulation, defective DNA damage response, aberrant cell cycle regulation, dysregulated prosurvival pathways), which were identified and described over the last years  [5, 10, 17]. The patient-specific T-PLL cancer cell samples of the first cohort were treated with eight drugs (bendamustine [alkylating agent], cladribine [ribonucleotide reductase inhibitor with additional epigenetic mode of action], dinaciclib [CDK inhibitor], ibrutinib [BTK/ITK inhibitor], idasanutlin [MDM2 inhibitor], romidepsin [HDAC2 inhibitor], ruxolitinib [JAK inhibitor], venetoclax [BCL2 inhibitor]) using seven different doses. The patient-specific samples of the second cohort were treated with six drugs (belinostat [HDAC inhibitor], bendamustine, cladribine, fludarabine [DNA synthesis inhibitor], KRT-232 [MDM2 inhibitor], venetoclax) using five doses. Both cohorts had three drugs in common: bendamustine, cladribine and venetoclax. Three conventional cytostatic drugs (bendamustine, cladribine, fludarabine) were among the tested drugs. Details to the drugs are summarized in Supplementary Table S1 and the drug response profiles are provided in Supplementary Table S2. The age distributions of both cohorts were similar (U-test: *p* = 0.55, median age: 74.5 years cohort 1 and 76 years for cohort 2). The female to male ratio with 11:5 for the first cohort and 8:10 for the second cohort did not significantly differ (Fisher’s exact test: *p* = 0.19).

To show which patient-specific T-PLL cancer samples responded effectively to specific drugs, Table 1 highlights which drugs were able to reduce the percentage of surviving cultured peripheral blood mononuclear cells below 50% with respect to the underlying drug dosage schemes. Patient-specific T-PLL samples whose cell survival was going below 50% in the drug response curve are marked with a green ‘v’ and those T-PLL samples whose drug response curve did not reach a reduction below 50% are marked by a red ‘x’ for each specific drug. Further, all patients of both cohorts were assigned to one of the three specific T-PLL gene expression subgroups revealed in our previous study  [18] utilizing their gene expression profiles. The observed subgroup distribution of both cohorts was similar to those of the initial study (Table 1; T-PLL cohort 1: 5 SG1, 2 SG2, 9 SG3; T-PLL cohort 2: 6 SG1, 1 SG2, 11 SG3).

**Fig. 2:**
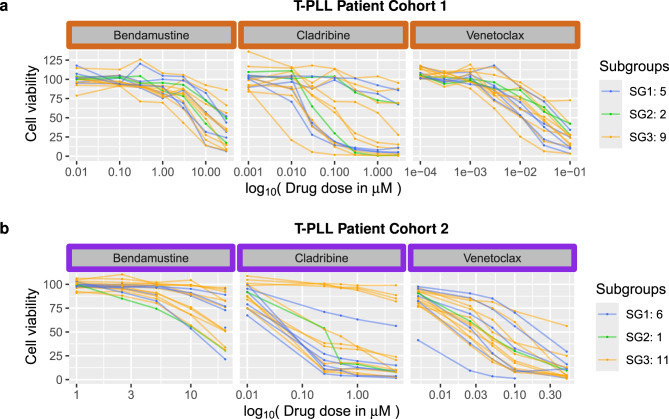
Drug response profiles of both considered T-PLL cohorts for the three overlapping drugs. The cell viability of cultured peripheral blood mononuclear cells of each T-PLL patient was quantified as percentage in relation to the negative control of untreated cells. The drug response profiles of the patient-specific samples are colored according to their T-PLL gene expression subgroup assignment (SG1: blue, SG2: green, SG3: orange). Dots within each curve highlight at which doses in micromolar (x-axis) measurements of the cell viabilities were taken (y-axis). Higher drug doses clearly reduced cell viabilities in most cases. Cells were growing in some cases at lower drug doses leading to cell viabilities greater than 100%

To characterize similarities and differences of the drug screens, the drug response profiles of the T-PLL cancer samples of individual patients are shown in Fig. 2 for the overlapping drugs of both cohorts. In addition, Supplementary Fig. S2 shows the drug response profiles for drugs that were only tested in one of both cohorts. Overall, the T-PLL cancer cell samples of most patients responded to most of the drugs, but there were also three T-PLL patients whose T-PLL cancer cell samples were more resistant to the majority of drugs (Table 1: p1375, p1401, p1755). Further, the drug response profiles in Supplementary Fig. S2 together with the patient-specific data in Table 1 clearly indicate that the two drugs ibrutinib and ruxolitinib did not strongly reduce the viability of T-PLL cancer cells for T-PLL samples of most patients. In addition, bendamustine was much more effective in the first than in the second cohort (Table 1, Fig. 2).

**Fig. 3:**
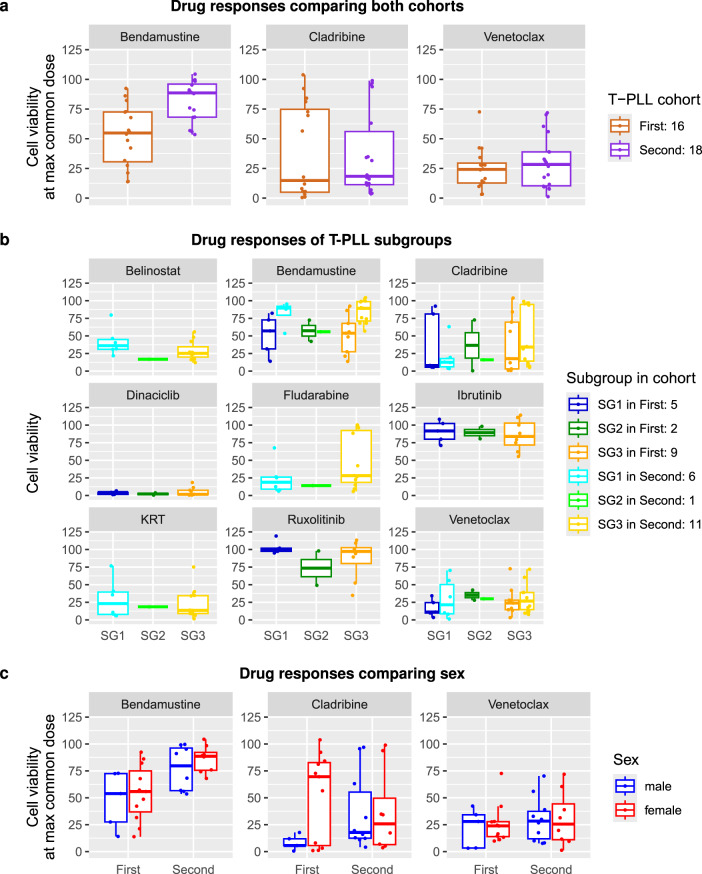
Comparison of drug response behavior of both considered T-PLL cohorts. a, Box plots of cell viabilities of the cultured peripheral blood mononuclear cells of the T-PLL patients measured at the maximal (highest) common drug dose of both cohorts: bendamustine $- 10\,{\mu}\text{M}$, cladribine $-1\,{\mu}\text{M}$, venetoclax $-0.1\,{\mu}\text{M}$. b, Box plots of cell viabilities stratified according to the assignments of patients to known T-PLL gene expression subgroups. For the three drugs bendamustine, cladribine, and venetoclax tested in both cohorts, cell viabilities at the highest common drug dose are shown and for the other six drugs that were only tested in one cohort cell viabilities at the highest drug dose are shown. c, Box plots of cell viabilities stratified according to sex of patients

Because of the similar sex ratio, the similar age distribution and the partially overlapping drug doses (Table 1, Supplementary Fig. S1), it was possible to utilize both cohorts for an additional combined comparative drug effectivity analysis. Considering the three overlapping drugs, bendamustine was more effective for T-PLL samples of patients in the first cohort (12 responders vs. 4 non-responders) than in the second cohort (4 responders vs. 14 non-responders). Cladribine was effective for the majority of T-PLL samples of patients of both cohorts (cohort 1: 10 responders vs. 6 non-responders, cohort 2: 13 responders vs. 5 non-responders). Overall, with respect to the underlying drug dosage schemes, the most effective drug to reduce the T-PLL cancer cell viability of T-PLL samples below 50% in both cohorts was venetoclax for which only one non-responder was observed in each cohort. Nevertheless, such a direct comparison of the response behavior of the three overlapping drugs across the two cohorts was not straight-forward, because of the different drug concentrations that were used in both drug response screens (Supplementary Fig. S1). Therefore, the cell viability observed at the highest common drug dose of both cohorts was considered to realize this comparison (Fig. 3a). No significant difference of the median cell viabilities was observed for both cohorts considering cladribine or venetoclax, but again bendamustine showed significantly different median cell viabilities between both cohorts (U-test: *p* = 0.0009 with a median of 54.9% for the first cohort and 88.6% for the second cohort). These findings for bendamustine were also confirmed considering the area under the drug response curve (AUC) for the longest common dose interval of each drug tested in both cohorts (Supplementary Fig. S12a, U-test: *p* = 0.0007 with a median AUC of 74 for the first cohort and 96 for the second cohort). Similar results were obtained when considering the median effective dose (ED50) to analyze the response behavior of the three overlapping drugs, whereas the area under the drug response curve (AUC) or the drug-specific sensitivity score (DSS) indicated differences between both cohorts for the three drugs most likely due to different drug dosages used in the underlying screens. These observed differences for other drug response measures were only significant for venetoclax (Supplementary Fig. S3).

### No difference in drug response behavior between T-PLL gene expression subgroups

The drug response curves of both cohorts in Fig. 2 and Supplementary Fig. S2 indicate that there is no difference in the response behavior between the three previously revealed T-PLL gene expression subgroups  [18] to which the individual T-PLL patients were assigned based on the gene expression profiles of their T-PLL cells. To further systematically compare the drug response behavior of the three subgroups for the nine tested drugs, the cell viability of the cultured peripheral blood mononuclear cells at the highest common dose was considered for each of the three overlapping drugs, whereas for drugs that were only tested in one of both cohorts the cell viability at the highest dose was considered (Fig. 3b). Generally, there was no significant difference in the cell viability across the three T-PLL gene expression subgroups for each considered drug (Kruskal-Wallis test: *p* > 0.05). This observation is also supported by the other drug response measures ED50, AUC, and DSS (Supplementary Fig. S4). Further, also the area under the drug response curve (AUC) for the longest common dose interval of each drug tested in both cohorts did not show significant difference in the response behavior of T-PLL gene expression subgroups (Supplementary Fig. S12b).

### No sex difference in drug response behavior of T-PLL patient samples

The two considered patient cohorts have a similar sex distribution with a female to male ratio of 11:5 for the first cohort and 8:10 for the second cohort (Fisher’s exact test: *p* = 0.19). This offered the possibility to test for potential differences in drug response between female and male patients. Overall, there were no significant differences in median cell viabilities comparing the T-PLL cell samples from female and male patients (Fig. 3c, Supplementary Fig. S5). This absence of sex differences in drug response was also confirmed considering the area under the drug response curve (AUC) for the longest common dose interval of each drug tested in both cohorts as alternative quality measure (Supplementary Fig. S12c). However, it should also be noted that independent of the sex for several of the tested drugs the responses of the patient samples were relatively heterogeneous ranging from very low to relatively high cell viability after treatment (e.g. bendamustine, cladribine, fludarabine, venetoclax).

### Gene expression and signaling pathway differences between potential drug-specific responders and non-responders

Since the cultured peripheral blood mononuclear cells of some T-PLL patients were more susceptible to some drugs than the cultured cells of other patients (e.g. Table 1, Fig. 2, Supplementary Fig. S2), we analyzed if the observed drug response differences were associated with the expression behavior of genes. Supported by the existence or non-existence of the drug-specific ED50 values for the T-PLL samples of patients, we assigned each patient either to the responder or to the non-responder group depending on the fact if the corresponding cell viability of its T-PLL sample dropped below 50%. Only three drugs (bendamustine, cladribine, fludarabine) could be considered for this exploratory analysis, because these drugs were effective for several or most T-PLL samples of patients but also contained at least four patients whose T-PLL sample did not respond well to the specific drug. The results of the three drug-specific differential gene expression analysis comparing the gene expression profiles of responders and non-responders are provided in Supplementary Table S3. Corresponding volcano plots are shown in Supplementary Fig. S6. It is important to note that the results of these drug-specific differential gene expression analyses should only be considered as an exploratory approach for gene and pathway hypothesis generation that requires additional experimentation beyond the performed annotation and literature review described in the remaining part of this section and in the next section.

**Fig. 4:**
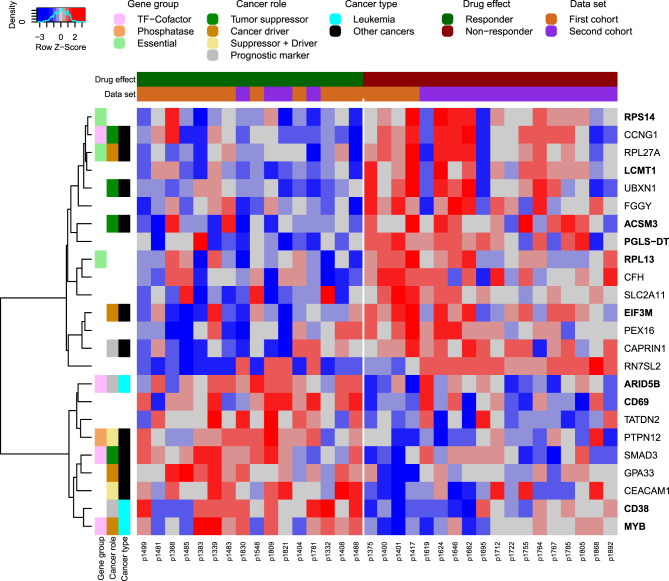
Heatmap of differentially expressed genes between potential responders and non-responders treated with bendamustine. All differentially expressed genes between T-PLL patients whose cultured peripheral blood mononuclear cells strongly responded or that did not strongly respond to bendamustine treatment are included up to a p-value cutoff of 0.01 (Supplementary Table S3). The gene expression data were obtained from the T-PLL patient samples prior to treatment independent of the drug response analysis. The individual cells of the heatmap represent z-scores of the log_2_-expression values of the genes across the patient samples scaled per row to emphasize gene-specific expression difference between responders and non-responders. The columns represent the T-PLL patient samples. The T-PLL samples are ordered according to their strength of drug response (drug effect bar above the heatmap: dark green potential responders ordered in ascending order of their ED50 values; brown: potential non-responders). The data set bar above the heatmap shows if a T-PLL patient was part of the first or the second T-PLL cohort. The rows of the heatmap that represent the genes were hierarchically clustered. Color coding bars on the left side of the heatmap highlight gene functions and known roles in cancer. Bold-marked genes on the right side of the heatmap were also differentially expressed at the p-value cutoff of 0.05 in each of both cohorts for separate cohort-specific differential gene expression analyses between potential responders and non-responders

Globally, almost none of the genes remained significant after correction for multiple testing  [47], because the transcriptomes of the T-PLL samples of the responders and non-responders were very similar in combination with the fact that only a relatively limited number of patients was available for each comparison. Nevertheless, a ranking of the differential expression potential of the genes by the obtained p-values coupled with a heatmap visualization of genes with p-values equal or less than 0.01 clearly indicate that some genes with discriminative potential between responders and non-responders exist for each drug. This is examplarily shown in Fig. 4 for bendamustine and visualized for cladribine in Supplementary Fig. S7 and for fludarabine in Supplementary Fig. S8.

In-depth gene function and literature analyses showed that several of the differentially expressed genes in the drug-specific heatmaps play important roles in leukemia or other types of cancer (Supplementary Table S4). This is visualized left to each heatmap and included known tumor suppressors (e.g. bendamustine: *ACSM3*, *CCNG1*, *SMAD3*; cladribine: *CELF2*, *MSRA*, *PPP3CC*; fludarabine: *CEP164*, *GNG7*, *PDLIM2*), known cancer drivers (e.g. bendamustine: *EIF3M*, *MYB*, *RPL27A*; cladribine: *SPOCK2*, *RNF13*, *PCM1*; fludarabine: *ALDOC*, *CD9*, *SMC4*), or genes known that they can have both of these roles (e.g. bendamustine: *CEACAM1*, *PTPN12*; cladribine: *DLEU1*, *GLI1*, *CCAR2*; fludarabine: *DLL1*, *GALNT6*, *MATK*). Several of these genes and also other genes in the drug-specific heatmaps frequently encode for transcription factors, kinases, phosphatases or genes essential for cell survival.

Further, an additional signaling pathway enrichment analysis of differentially expressed genes between bendamustine responders and non-responders at the p-value cutoff of 0.05 revealed that cell cycle and notch signaling were significantly enriched for genes with increased expression in the responder group (Supplementary Fig. S9a, Fisher’s exact test: FDR-adjusted *p* < 0.01). No significant signaling pathway enrichments were found for cladribine and fludarabine (Supplementary Fig. S9b,c). In addition, none of the known target genes of cladribine and fludarabine (Supplementary Table S1, Supplementary Fig. S6) were among the differentially expressed genes that distinguished responders from non-responders in the drug-specific analyses. Thus, other genes and factors potentially influence the drug-specific response behavior.

### Functional roles of differentially expressed genes distinguishing potential responders and non-responders

To get more detailed insights to cellular processes and molecular mechanisms that may contribute to the observed drug response differences between potential responders and non-responders, the differentially expressed genes predicted for the response behavior of the three drugs bendamustine, cladribine, and fludarabine at the p-value cutoff of 0.01 were further analyzed with help of GeneCards  [48], ATLAS of Genetics and Cytogenetics in Oncology and Haematology  [49], MalaCards  [50], and manual literature research using PubMed  [51] (Supplementary Table S4).

More than half of the differentially expressed genes between bendamustine responders and non-responders and more than one third of the differentially expressed genes found for the response behavior to cladribine or fludarabine were reported to be involved in different types of cancer. This included for example for bendamustine *EIF3M* involved in cell proliferation, cell cycle progression and cell death  [52], *SMAD3* involved in epithelial-to-mesenchymal transition, tumor suppressor and metastasis formation  [53], and *UBXN1* influencing EGFR signaling  [54] (Fig. 4). For cladribine, *NR2F6* involved in cell growth, migration and invasion  [55], *RNF139* involved in cell viability, invasion and AKT signaling  [56], and *SORBS3* involved in interleukin-6 signaling  [57] were found (Supplementary Fig. S7). For fludarabine, *DDX10*, *GMDS*, and *MAGI3*, which are all three involved in cell proliferation  [58–60], were included for example (Supplementary Fig. S8).

Moreover, about 10% of the differentially expressed genes are known to play a role in leukemia. This includes for example for bendamustine *ARID5B* associated with susceptibility of childhood acute lymphoblastic leukemia and treatment outcome  [61], *CD38* associated with cell survival and proliferation in chronic lymphocytic leukemia  [62], and the oncogene *MYB* involved in different leukemias  [63] (Fig. 4). For cladribine, *SAMHD1* involved in proliferation of acute myeloid leukemia cells  [64] and *PCM1* involved in gene fusions in atypical chronic myeloid leukemia  [65] were found (Supplementary Fig. S7). For fludarabine, *SMC4* associated with the outcome of pediatric acute lymphoblastic leukemia  [66], and *TNFRSF13C* and *HLA-DQB1* for which specific gene variants had been associated with different types of leukemia  [67, 68] were included (Supplementary Fig. S8).

Further, several of the differentially expressed genes predicted between the drug-specific responders and non-responders at the p-value cutoff of 0.01 overlapped for cladribine and fludarabine. This overlap comprised 17 genes that had the same direction of expression changes for both drugs (Supplementary Table S4, Supplementary Fig. S7, Supplementary Fig. S8). These 17 genes included 14 genes that were down-regulated in responders (*SLC9A3–AS1*, *LINC02762*, *TIRAP*, *NSG1*, *IRS1*, *MAP4K3-DT*, *FRY*, *RUNX2*, *CCDC50*, *CAMK1D*, *CCDC71L*, *ROBO3*, *PLEKHA7*, *NLGN2*) and three genes that were up-regulated in responders (*CD3E*, *MORC3*, *KLF8*). Four of these genes were reported for leukemias (*IRS1*  [69], *RUNX2*  [70], *CCDC50*  [71] and *ROBO3*  [72]) and four others were reported to be involved in other types of cancers (*NSG1*  [73], *CAMK1D*  [74], *CD3E*  [75], *KLF8*  [76]). These genes often play a role in immune response and the regulation of cancer-related signaling pathways.

In addition, there was no overlap of differentially expressed genes for the bendamustine-specific comparison of responders and non-responders and the differentially expressed genes predicted for the other two drugs. This difference might be expected, because cladribine and fludarabine have a similar mechanism of action as purine anti-metabolites, whereas bendamustine is an alkylating agent that directly damages DNA.

### Direct correlations between gene expression and drug response

To complement the previous exploratory differential gene expression analysis, correlations between gene-specific expression levels and corresponding patient-specific drug responses were computed for bendamustine, cladribine, and fludarabine. This correlation analysis avoids potential limitations of the classification of T-PLL samples as strongly or not strongly responding to a drug. Similar to the results of the differential gene expression analysis, no gene was significantly associated with the response to one of the three drugs after correction for multiple testing (Supplementary Table S8). Nevertheless, the top-ranking genes showed again relatively strong positive or negative correlations between their expression and patient-specific drug responses for all three drugs (Supplementary Fig. S13). In addition, especially for cladribine and fludarabine, a large overlap of the highly correlated genes with the previously predicted differentially expressed genes was observed, whereas the overlap was clearly less for bendamustine (bendamustine: 6 of 41 (14.6%), cladribine: 69 of 221 (31.2%), and fludarabine: 52 of 136 (38.2%) for *p* < 0.01, Supplementary Fig. S14). Many of these overlapping genes are known to play important roles in cancer and some of them were already reported in relation to leukemia (Supplementary Fig. S14, leukemia-associated genes: *SP140* for bendamustine; *SAMHD1*, *CCAR2*, *PCM1*, *PP1R14B*, *SP140*, *PDLIM2* for cladribine; *RUNX2*, *COIL*, *SP140*, *PDLIM2* for fludarabine). Especially, the two tumor suppressors *SP140* predicted for all three drugs and *PDLIM2* predicted for cladribine and fludarabine could be of greater general interest for future experiments.

### Comparison of the drug response behavior of the two analyzed T-PLL patient cohorts to a third validation cohort

Another drug response screen for T-PLL was published by Andersson et al. (2018) including 12 T-PLL patients  [34]. The T-PLL diagnosis criteria, the cell extraction techniques, and the drug response measurement platform were the same as those used for our two cohorts. The Andersson screen contains eight drugs that were also considered in at least one of our two cohorts (belinostat, bendamustine, cladribine, dinaciclib, fludarabine, ibrutinib, ruxolitinib, venetoclax). The drug dosage schemes were similar across the three screens (Supplementary Fig. S1). Further, seven T-PLL patients of the Andersson screen were also included in our first cohort (p1339, p1368, p1375, p1400, p1401, p1404, p1417). Thus, the drug response data of this third cohort represented a good basis for a validation of findings form our two T-PLL patient cohorts.

**Fig. 5:**
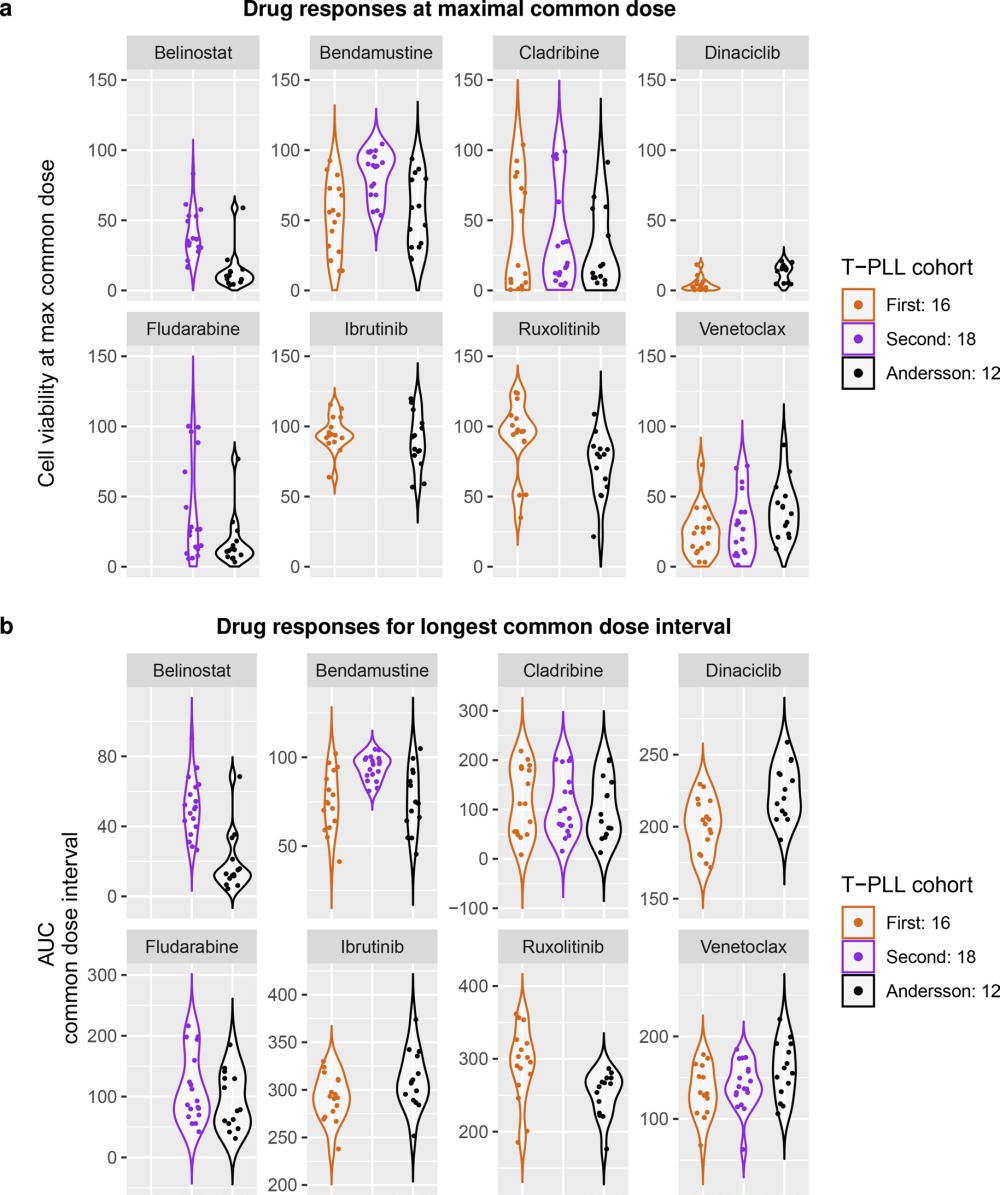
Comparison of the drug response screens of the two main T-PLL cohorts to the Andersson screen. a, Violin plots show the cell viabilities of the cultured peripheral blood mononuclear cells of the T-PLL patients measured at the maximal (highest) common drug dose of the compared screens (Supplementary Fig. S1). Individual points highlight the cell viabilities of the underlying T-PLL patient samples. Significant differences of median cell viabilities were observed for belinostat, bendamustine, dinaciclib, and ruxolitinib (U-test for two screens or kruskal-wallis test for three screens per drug: $p \le 0.01$). b, Violin plots similar to those in subpanel a but now representing the area under the drug response curve (AUC) for the longest common dose interval of a specific drug measured in two or three T-PLL cohorts. Significant differences were confirmed for the same four drugs as in subpanel a

The measured drug-specific responses of the T-PLL patient samples of the three drug response screens are shown in Fig. 5 and Supplementary Fig. S10. Significant differences of cell viabilities were found for four of eight drugs comprising belinostat, bendamustine, dinaciclib, and ruxolitinib (Fig. 5a: $p \le 0.01$ for Kruskal-Wallis test when a drug was tested in all three screens or U-test when a drug was only tested in two screens). These findings were also confirmed considering the area under the drug response curve (AUC) for the longest common dose interval of a specific drug measured in two or three T-PLL cohorts as an alternative measure of cell viability (Fig. 5b). Further, the drug response behavior of the seven shared patients of the Andersson screen and our first cohort were compared. Considering the six overlapping drugs between the two cohorts, the shared T-PLL patient samples showed highly similar responses for bendamustine, cladribine, and ibrutinib in both screens, whereas for venetoclax, dinaciclib, and ruxolitinib certain differences in the responses were observed (Supplementary Fig. S11). However, only dinaciclib showed significant differences for cell survival in both cohorts (Wilcoxon signed-rank test: *p* = 0.03125). Overall, the sample of the T-PLL patient p1375 showed the greatest response differences between both screens.

Globally, all these comparisons indicate that the responses to some of the tested drugs can be heterogeneous depending on the specific screen. Further, the relatively few T-PLL samples analyzed in each screen and their inclusion criteria may have contributed to the observed drug-specific differences. However, the responses to bendamustine, cladribine, and ibrutinib were highly reproducible for six of the seven shared patients between the Andersson screen and our first cohort.

## Discussion

Molecular mechanisms that contribute to T-PLL development were understood in greater detail over the last years due to the availability of genomewide molecular characterizations of T-PLL patients at different omics layers  [17]. This also contributed to the identification of new potential therapeutic T-PLL targets  [5, 10]. Such new targets could help to overcome the limitation of alemtuzumab as current major first-line therapy, which does not enable a long-term disease control. To address this, a platform to computationally predict and to experimentally test suited drug combinations for individual T-PLL patients was developed some years ago  [77]. To target specific pathomechanisms, preclinical data were already measured in a single-agent based drug screen  [34] and in a drug combination screen  [38] for samples from T-PLL patients.

So far, these drug response analyses were done without considering that the included T-PLL patients can be assigned to three different T-PLL gene expression subgroups  [18]. Since a large subset of the T-PLL patients from Schrader et al.  [17], which formed the basis of our prediction of T-PLL gene expression subgroups  [18], were also part of the recent drug response screen by von Jan et al.  [38], the major goal of our study here was to analyze if T-PLL samples of the patients from the three revealed T-PLL gene expression subgroups potentially differ in their response to specific drugs.

To realize this, we analyzed single-agent based drug screens of 34 T-PLL patients, which were provided by the Mustjoki laboratory in Helsinki and by the Herling laboratory in Cologne. The drug response profiles of 23 T-PLL patients (all 16 of our first and 7 of our second cohort) were already part of the joint drug response study of both laboratories  [38]. The two considered T-PLL cohorts represented treatment naive patients. The first cohort comprised 16 patients from Helsinki and the second cohort contained 18 patients from Cologne. The T-PLL samples of these 34 patients were analyzed for their response to a set of in total 11 drugs of which three tested drugs (bendamustine, cladribine, venetoclax) overlapped between both cohorts. The patient characteristics of both cohorts were comparable and did not significantly differ in the age distribution, the sex distribution, or the distribution of T-PLL gene expression subgroups.

Globally, we did not find any significant difference in the drug response behavior for patients of the three T-PLL gene expression subgroups considering different drug response measures. Thus, at least for the considered drugs the response of T-PLL samples did not depend on their underlying T-PLL gene expression subgroup, which suggests that these drugs could be considered in additional studies without the need to account for gene expression subgroups. In addition, there was also no difference when we compared the drug responses of T-PLL samples of male and female patients. Both observations are important results that indicate that especially the effective drugs (cladribine, venetoclax, dinaciclib, idasanutlin, romidepsin, KRT-232) among the tested drugs might potentially be suited to treat a broad range of T-PLL patients.

In more detail, considering the drug response behavior of all tested drugs, we found that ibrutinib (BTK/ITK inhibitor targeting B-cell receptor signaling) and ruxolitinib (JAK inhibitor targeting the JAK-STAT pathway) were not suited to strongly reduce the cell viability of T-PLL cancer cells with respect to the underlying dosage schemes. These results are in good accordance with the response of T-cell leukemia/lymphoma cell lines to treatments with ibrutinib and ruxolitinib  [38]. Ibrutinib had been found to enhance the sensitivity of T-PLL cells to venetoclax  [78, 79], but more recent results show that other drug combinations are more effective  [38]. The resistance to ruxolitinib could be associated with the activity and the mutation state of the JAK-STAT signaling pathway and a highly activating *STAT5B* mutation  [34]. Further, the conventional cytostatic agent cladribine (ribonucleotide reductase inhibitor with additional epigenetic mode of action) was effective in reducing the cell viability of T-PLL cancer cells for both cohorts, whereas the conventional cytostatic agent bendamustine (alkylating agent) was much more effective in the first than in the second T-PLL cohort. This strong batch effect observed for bendamustine was most likely triggered by differences in the bendamustine dosage schemes of both cohorts. The maximal common bendamustine dose shared between both cohorts was too low for several patient samples (especially for the second cohort) to strongly reduce the cell viability. Bendamustine responses of about half of the T-PLL samples of the second cohort were greater for larger doses, but these doses were not shared with the first cohort. Also general differences between the first cohort from Helsinki and the second cohort from Cologne may have contributed to the observed differences. The greater efficacy of cladribine is most likely driven by its additional epigenetic mode of action  [80], which may interfere with the known deregulation of major epigenetic regulators in T-PLL  [17]. Further, the sensitivity to cladribine is correlated with the expression of its target *RRM2*  [38]. Overall, with respect to the dosage schemes of the different drugs, the most effective drug to reduce the viability of T-PLL cancer cells was venetoclax (BCL2 inhibitor inducing apoptosis), which was tested for both analyzed T-PLL cohorts. However, combinations of venetoclax with other drugs had so far only shown limited clinical success for T-PLL patients  [29, 31, 32] and moderate efficacy in preclinical screens  [38]. Only T-PLL patients with a strong BCL2 dependency may profit from venetoclax. In addition, dinaciclib (CDK inhibitor), idasanutlin (MDM2 inhibitor inhibiting cell proliferation and inducing apoptosis), and romidepsin (HDAC inhibitor) were also very effective to reduce the T-PLL cancer cell viability, but these three drugs were only tested for the first T-PLL cohort. KRT-232 (MDM2 inhibitor inducing apoptosis) only tested in the second T-PLL cohort showed a similar effectivity to reduce the T-PLL cancer cell viability. Most of these effective drugs were also already tested for related T-cell leukemias. For example, dinaciclib was reported to be an effective drug in preclinical settings of T-cell acute lymphoblastic leukemia  [81]. Preclinical data showed that idasanutlin in combination with navitoclax induces apoptotic cell death in T-cell acute lymphoblastic leukemia  [82]. A newly developed romidepsin nanoparticle was recently reported to have improved efficacy in preclinical models of T-cell lymphoma  [83].

We further analyzed the gene expression profiles of treatment naive T-PLL samples from patients whose T-PLL samples did or did not strongly respond to specific drugs. These analyses extend a similar analysis that was done for cladribine in  [38] for less patients. The corresponding differential gene expression analyses were possible for three drugs (bendamustine, cladribine, fludarabine), whose drug response profiles enabled to distinguish between strongly responding and not strongly responding T-PLL samples. Overall, responding and non-responding T-PLL samples had very similar gene expression profiles, but a ranking of genes based on their discriminative potential between responders and non-responders still allowed to predict genes that were able to distinguish both response groups. Many of the top-ranking genes predicted by the differential gene expression analysis were also confirmed by an additional direct correlation analysis between gene expression and drug response. Several of the differentially expressed genes are known to play a role in leukemia or other types of cancer. In accordance with their mechanism of action as purine anti-metabolites, an overlap of 17 differentially expressed genes was found between cladribine and fludarabine. Several of these differentially expressed genes were known cancer genes involved in the regulation of immune response and cancer signaling pathways, but none of the known targets of both drugs were among the differentially expressed genes between potential responders and non-responders. This indicates that additional factors among the differentially expressed genes may contribute to the response behavior of T-PLL patient samples to cladribine and fludarabine.

Finally, we compared the drug response behavior of our two T-PLL cohorts to a third related T-PLL cohort from Andersson et al. (2018)  [34]. This revealed significant differences for four of the eight overlapping drugs (belinostat, bendamustine, dinaciclib, ruxolitinib) considering all T-PLL patients. Thus, responses to specific drugs can be heterogeneous and more patients would have been required within each cohort to better cover the broad spectrum of patient-specific drug responses. However, when we focused on the seven shared patients between the Andersson cohort and our first T-PLL cohort only the significant difference for dinaciclib remained. This clearly indicates that the patient-specific drug response profiles are highly reproducible between both cohorts, which supports and strengthens our findings.

Nevertheless, it is also important to note that our study has some limitations. The two main T-PLL drug response cohorts and the third validation cohort that we compared are relatively small. Further, only three drugs were overlapping between the two main cohorts. This was partially compensated by the comparison to the validation cohort, which shared six drugs with the first cohort and five drugs with the second cohort. In addition, the drug response data of the three cohorts were measured based on different drug dosage schemes, which complicated the direct comparisons and only allowed us to perform direct comparisons of drug responses based on the maximal common dose or the longest common shared dose interval. Further, the exploratory differential gene expression analyses between T-PLL samples that strongly responded to those that did not strongly respond to specific drugs lack statistical power and their interesting biological indications should only be considered as explorative basis to determine hypotheses for future studies. The same applies to the direct correlation analysis between gene expression levels and drug responses. However, most of these limitations can hopefully be overcome in a future study based on a larger T-PLL cohort. All samples should be analyzed based on the same drug-specific dosage schemes on the same platform and corresponding transcriptomes of samples should be measured on only one instead of different platforms.

## Conclusions

Our study contributes to a better characterization of the therapeutic potential of specific drugs for T-PLL in relation to known T-PLL gene expression subgroups. None of the responses to the tested drugs were associated with the underlying T-PLL gene expression subgroups. This finding is important for future preclinical drug response studies and may ease the design of potential clinical trials. In addition, our performed differential gene expression analysis between T-PLL samples that strongly responded to a specific drug to those that did not strongly respond could help to further stratify patients and may contribute to better understand which molecular settings are required to obtain more effective responses.

## Electronic supplementary material

Below is the link to the electronic supplementary material.

Supplementary Material 1

Supplementary Material 2

Supplementary Material 3

Supplementary Material 4

Supplementary Material 5

Supplementary Material 6

Supplementary Material 7

Supplementary Material 8

Supplementary Material 9

Supplementary Material 10

Supplementary Material 11

Supplementary Material 12

Supplementary Material 13

Supplementary Material 14

Supplementary Material 15

Supplementary Material 16

Supplementary Material 17

Supplementary Material 18

Supplementary Material 19

Supplementary Material 20

Supplementary Material 21

Supplementary Material 22

Supplementary Material 23

## Data Availability

All analyzed drug response profiles are provided in Supplementary Table S2. All considered gene expression profiles are provided in Supplementary Table S5. All analyses were performed in R using standard packages and functions described in the Methods section. The implemented R scripts and corresponding data sets are publicly available from Zenodo at https://doi.org/10.5281/zenodo.16942206.

## Abbreviations

T-PLL: T-prolymphocytic leukemia
FDR: False discovery rate
