# Supplementary material for: Comparative preclinical drug response analyses of T-prolymphocytic leukemia reveal no differences between known gene expression subgroups
Source: Biol Direct. 2025 Oct 27;20:106. doi: 10.1186/s13062-025-00701-3 (PMC12557856; doi:10.1186/s13062-025-00701-3)
Supplement: Supplementary file 1 — Supplementary Material 1 [file 13062_2025_701_MOESM1_ESM.pdf]

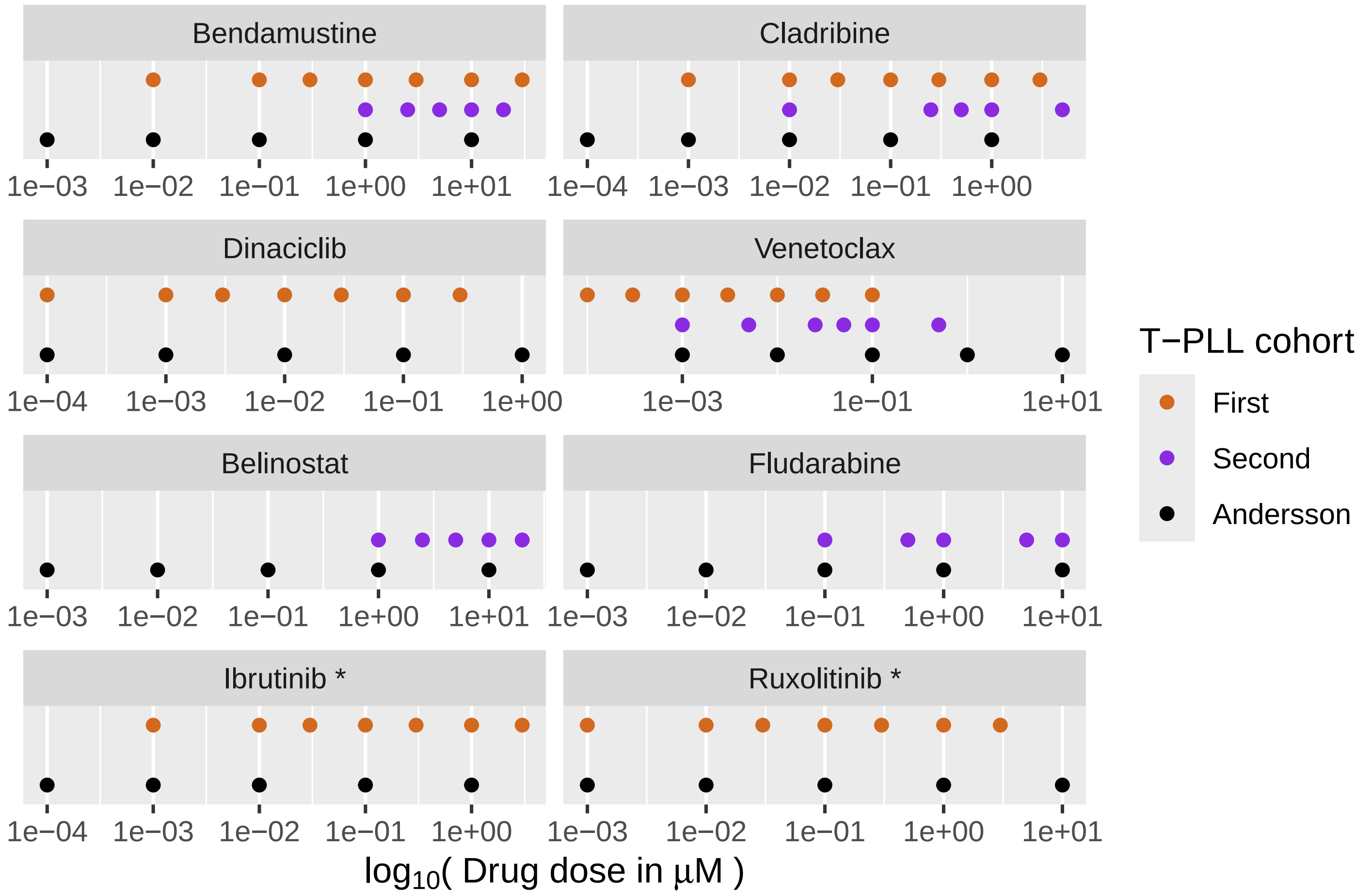

Figure S1: Overview of drug dosage overlaps between the three considered T-PLL patient cohorts. Drugs marked with ’*’ were not effective for samples of T-PLL patients (see Table 1 in main manuscript).
