# Supplementary material for: Comparative preclinical drug response analyses of T-prolymphocytic leukemia reveal no differences between known gene expression subgroups
Source: Biol Direct. 2025 Oct 27;20:106. doi: 10.1186/s13062-025-00701-3 (PMC12557856; doi:10.1186/s13062-025-00701-3)
Supplement: Supplementary file 2 — Supplementary Material 2 [file 13062_2025_701_MOESM2_ESM.pdf]

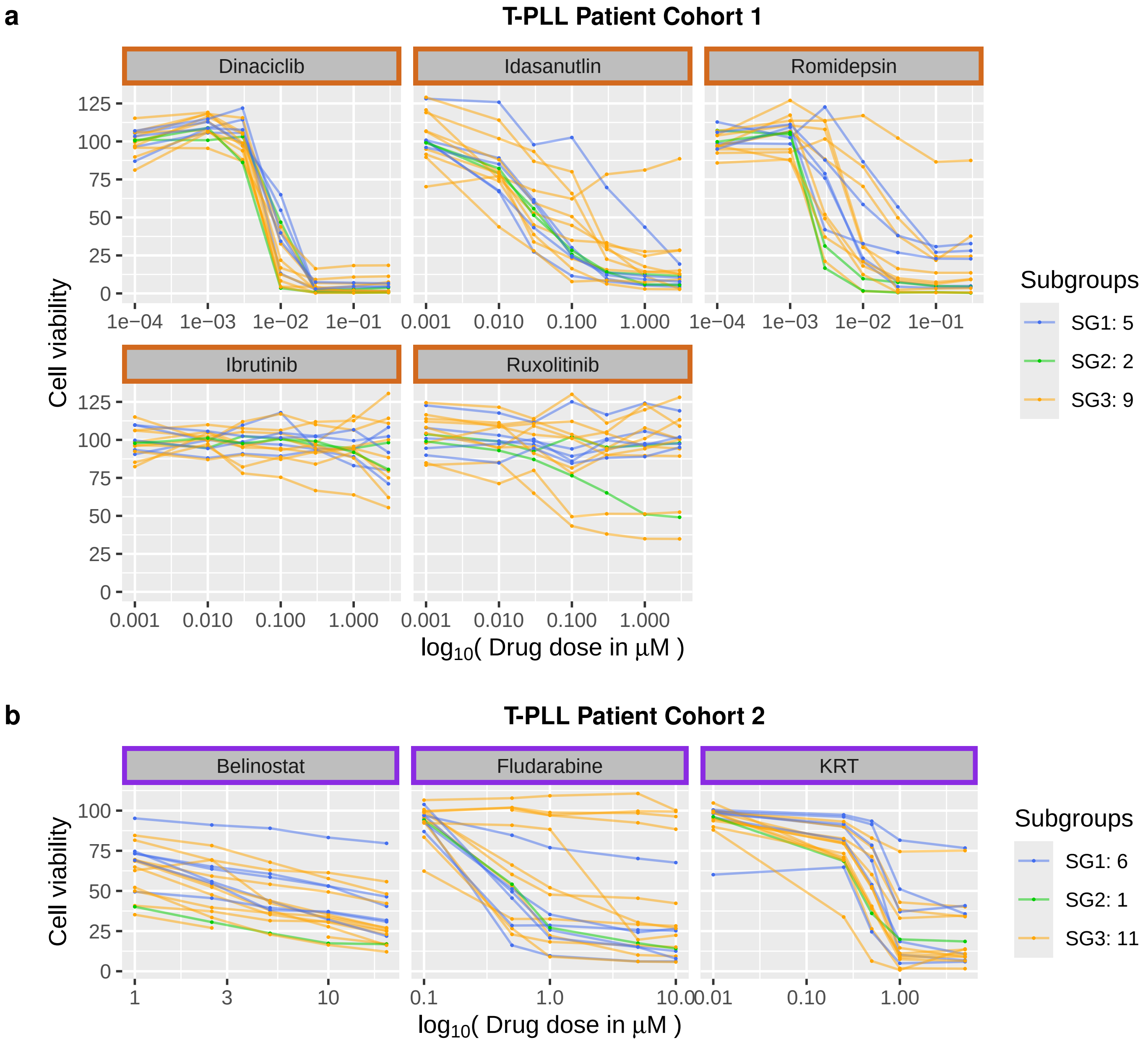

Figure S2: Drug response profiles of both considered T-PLL cohorts for drugs that were only tested in one of both cohorts. The cell viability of cultured peripheral blood mononuclear cells of each T-PLL patient was quantified as percentage in relation to the negative control of untreated cells. The drug response profiles of the patient-specific samples are colored according to their T-PLL gene expression subgroup assignment (SG1: blue, SG2: green, SG3: orange). Dots within each curve highlight at which doses in micromolar (x-axis) measurements of the cell viabilities were taken (y-axis).
