# Supplementary material for: Comparative preclinical drug response analyses of T-prolymphocytic leukemia reveal no differences between known gene expression subgroups
Source: Biol Direct. 2025 Oct 27;20:106. doi: 10.1186/s13062-025-00701-3 (PMC12557856; doi:10.1186/s13062-025-00701-3)
Supplement: Supplementary file 3 — Supplementary Material 3 [file 13062_2025_701_MOESM3_ESM.pdf]

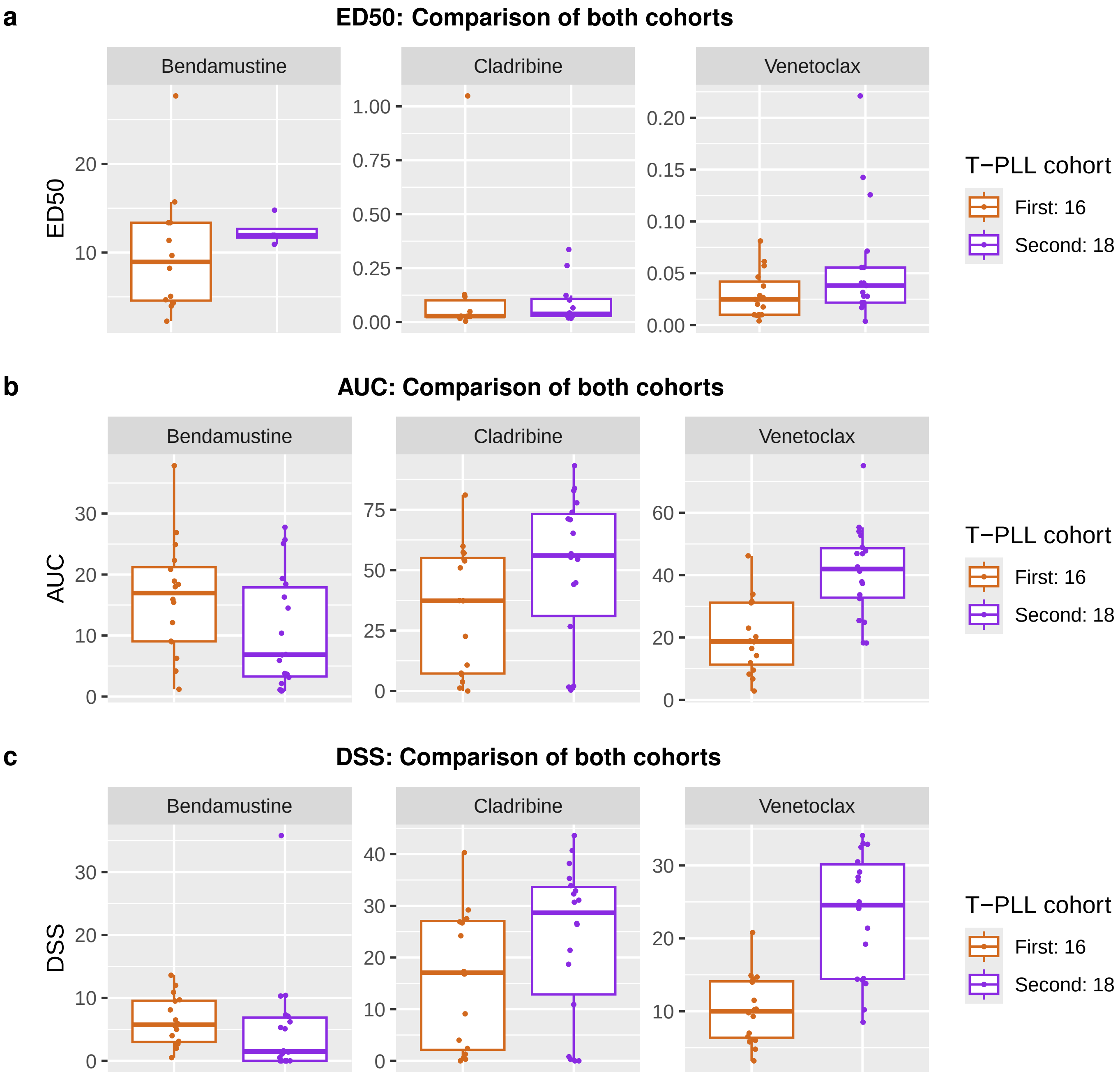

Figure S3: Comparison of drug response behavior of both considered T-PLL cohorts considering additional quality measures. See methods section of the main manuscript for details to the computation of ED50 (median effective dose), AUC (area under the drug response curve), and DSS (drug-specific sensitivity score). The box plots of the ED50 values (a) confirm our findings for the cell viabilities in Figure 3a of the main manuscript, whereas for AUC (b) and DSS (c) stronger drug response shifts between the two T-PLL cohorts exist. One factor that most likely also contributes to the observed shifts is the different drug dosage scheme used for both cohorts (Figure S1). However, U-tests only confirmed significant median shifts for venetoclax for AUC and DSS at the significance level of 5%, whereas the other shifts were not significant.
