# Supplementary material for: Comparative preclinical drug response analyses of T-prolymphocytic leukemia reveal no differences between known gene expression subgroups
Source: Biol Direct. 2025 Oct 27;20:106. doi: 10.1186/s13062-025-00701-3 (PMC12557856; doi:10.1186/s13062-025-00701-3)
Supplement: Supplementary file 4 — Supplementary Material 4 [file 13062_2025_701_MOESM4_ESM.pdf]

a ED50: Comparison of T-PLL subgroups for both cohorts

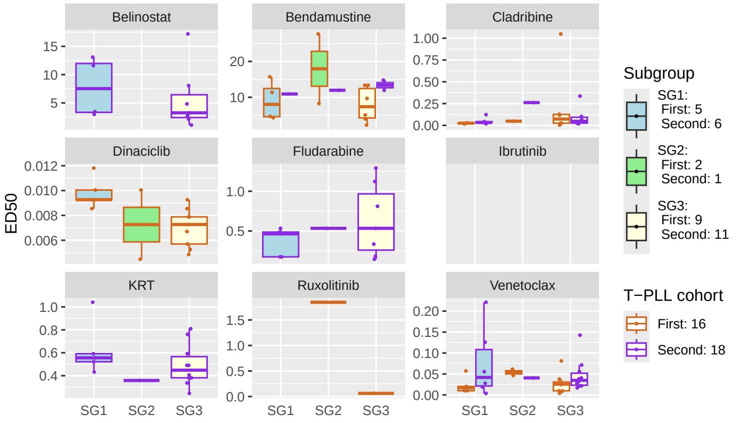

b AUC: Comparison of T-PLL subgroups for both cohorts

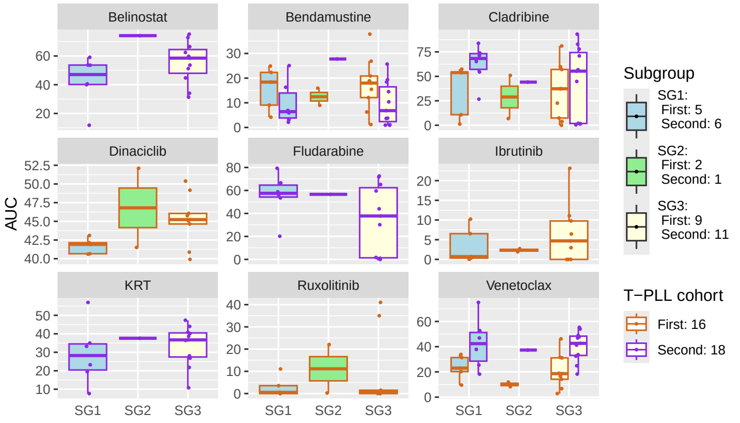

c DSS: Comparison of T-PLL subgroups for both cohorts

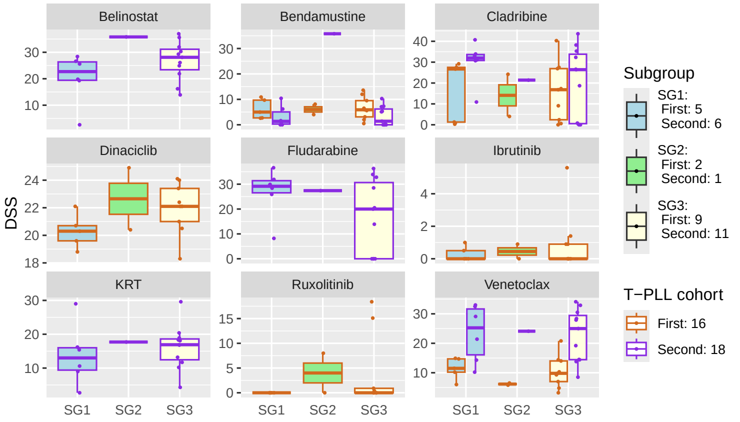

Figure S4: Comparison of drug response behavior of T-PLL gene expression subgroups for both considered T-PLL cohorts considering additional quality measures. See methods section of the main manuscript for details to the computation of ED50 (median effective dose), AUC (area under the drug response curve), and DSS (drug-specific sensitivity score). Overall, only one apparent difference is found for ED50 for dinaciclib (Kruskal-Wallis test, $p = 0.046$), but after correction for multiple testing this difference was also not significant anymore.
