# Supplementary material for: Comparative preclinical drug response analyses of T-prolymphocytic leukemia reveal no differences between known gene expression subgroups
Source: Biol Direct. 2025 Oct 27;20:106. doi: 10.1186/s13062-025-00701-3 (PMC12557856; doi:10.1186/s13062-025-00701-3)
Supplement: Supplementary file 5 — Supplementary Material 5 [file 13062_2025_701_MOESM5_ESM.pdf]

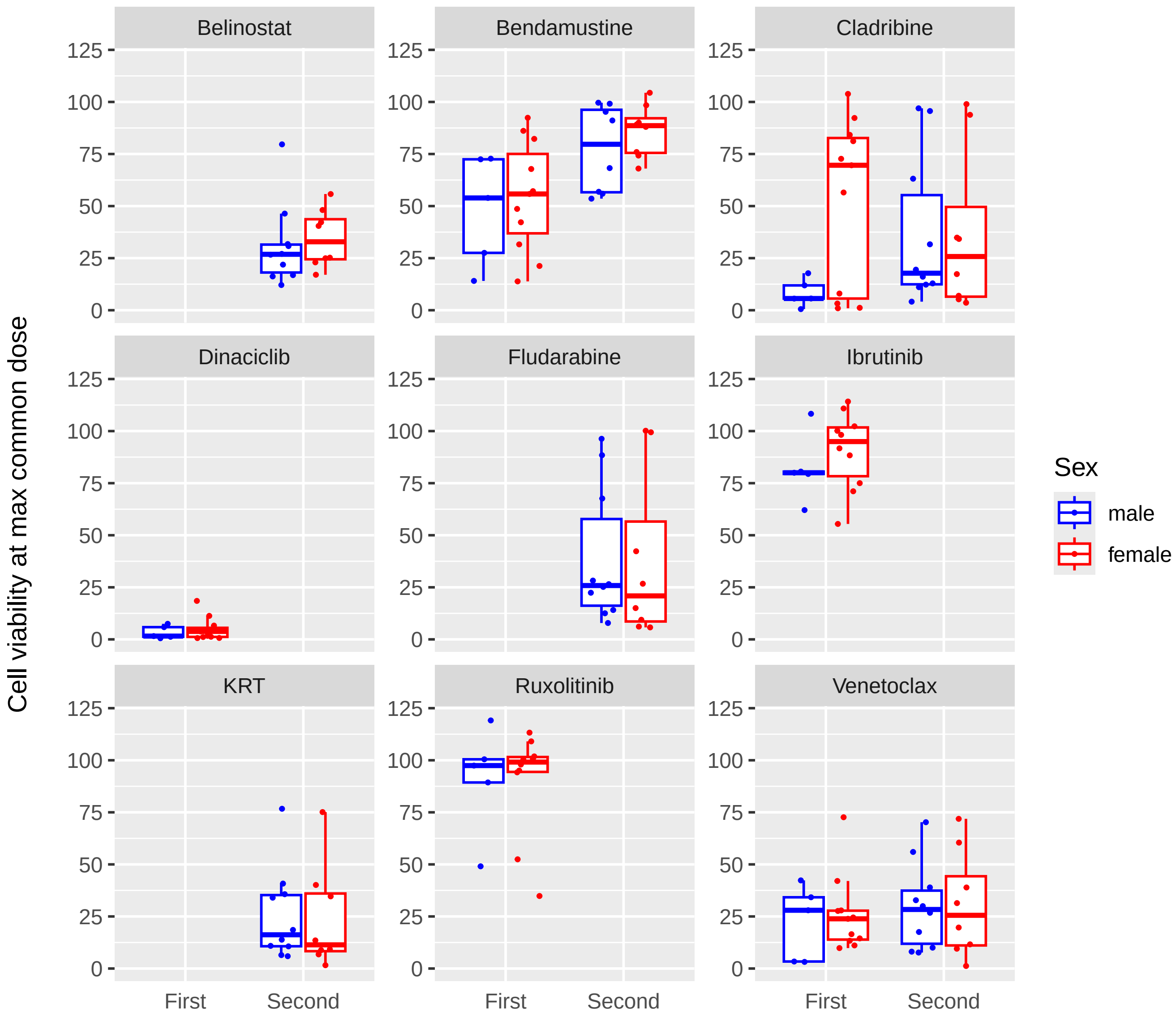

Figure S5: Comparison of sex-specific drug response behavior for both considered T-PLL cohorts. Shown are box plots of cell viabilities of cultured peripheral blood mononuclear cells of each T-PLL patient at the maximal common dose between the first and the second T-PLL cohort (Figure S1). The female to male ratio is 11:5 for the first cohort and 8:10 for the second cohort. No significant differences of minimal cell viabilities were found between female and male patients for each tested drug (U-tests: $p > 0.05$).
