# Supplementary material for: Comparative preclinical drug response analyses of T-prolymphocytic leukemia reveal no differences between known gene expression subgroups
Source: Biol Direct. 2025 Oct 27;20:106. doi: 10.1186/s13062-025-00701-3 (PMC12557856; doi:10.1186/s13062-025-00701-3)
Supplement: Supplementary file 6 — Supplementary Material 6 [file 13062_2025_701_MOESM6_ESM.pdf]

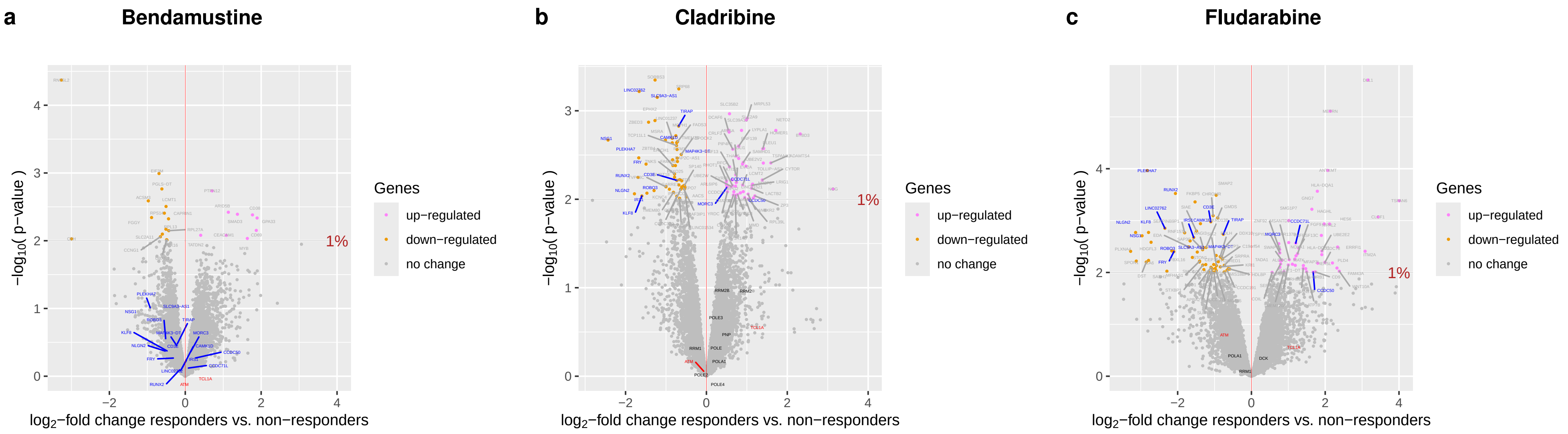

Figure S6: Volcano plots of differential gene expression analysis between responders and non-responders for the three drugs bendamustine (a), cladribine (b), and fludarabine (c). Different genes are labeled: red - T-PLL-specific genes, black - known drug-specific target genes, blue - common differentially expressed genes for cladribine and fludarabine with $p \leq 0.01$, and grey - drug-specific differentially expressed genes with $p \leq 0.01$.
