# Supplementary material for: Comparative preclinical drug response analyses of T-prolymphocytic leukemia reveal no differences between known gene expression subgroups
Source: Biol Direct. 2025 Oct 27;20:106. doi: 10.1186/s13062-025-00701-3 (PMC12557856; doi:10.1186/s13062-025-00701-3)
Supplement: Supplementary file 8 — Supplementary Material 8 [file 13062_2025_701_MOESM8_ESM.pdf]

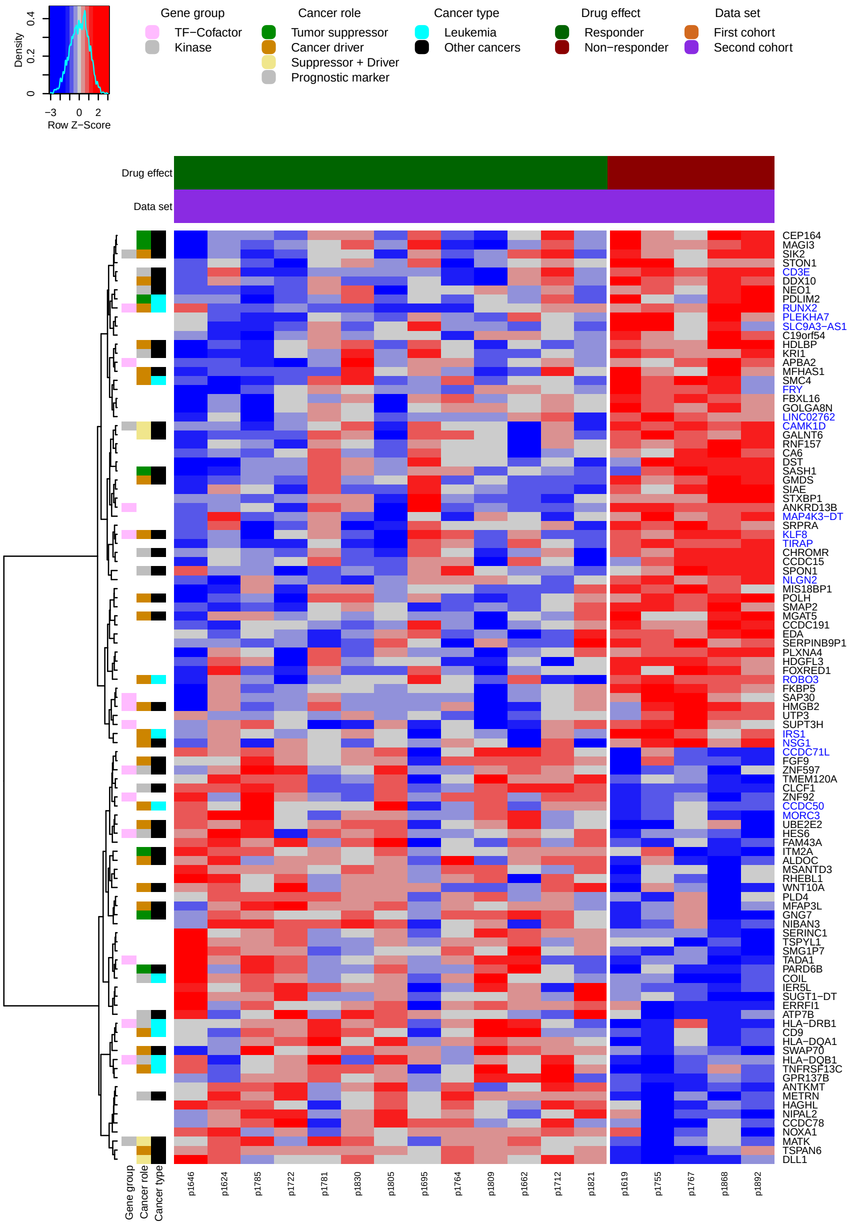

Figure S8: Heatmap of differentially expressed genes between potential responders and non-responders treated with fludarabine. All differentially expressed genes between T-PLL patients whose cultured peripheral blood mononuclear cells strongly responded or that did not strongly respond to fludarabine treatment are included up to a p-value cutoff of 0.01 (Table S3). The gene expression data were obtained from the T-PLL patient samples prior to treatment independent of the drug response analysis. The individual cells of the heatmap represent z-scores of the $\log_2$-expression values of the genes across the patient samples scaled per row to emphasize gene-specific expression difference between responders and non-responders. The columns represent the T-PLL patients. The potential responders are ordered according to their strength of drug response (drug effect bar above the heatmap: dark green potential responders ordered in ascending order of their ED50 values; brown: potential non-responders). The data set bar above the heatmap shows if a T-PLL patient was part of the first or the second T-PLL cohort. The rows of the heatmap that represent the genes were hierarchically clustered. Color coding bars on the left side of the heatmap highlight gene functions and known roles in cancer. Gene names colored in blue represent differentially expressed genes overlapping between cladribine and fludarabine.
