# Supplementary material for: Comparative preclinical drug response analyses of T-prolymphocytic leukemia reveal no differences between known gene expression subgroups
Source: Biol Direct. 2025 Oct 27;20:106. doi: 10.1186/s13062-025-00701-3 (PMC12557856; doi:10.1186/s13062-025-00701-3)
Supplement: Supplementary file 9 — Supplementary Material 9 [file 13062_2025_701_MOESM9_ESM.pdf]

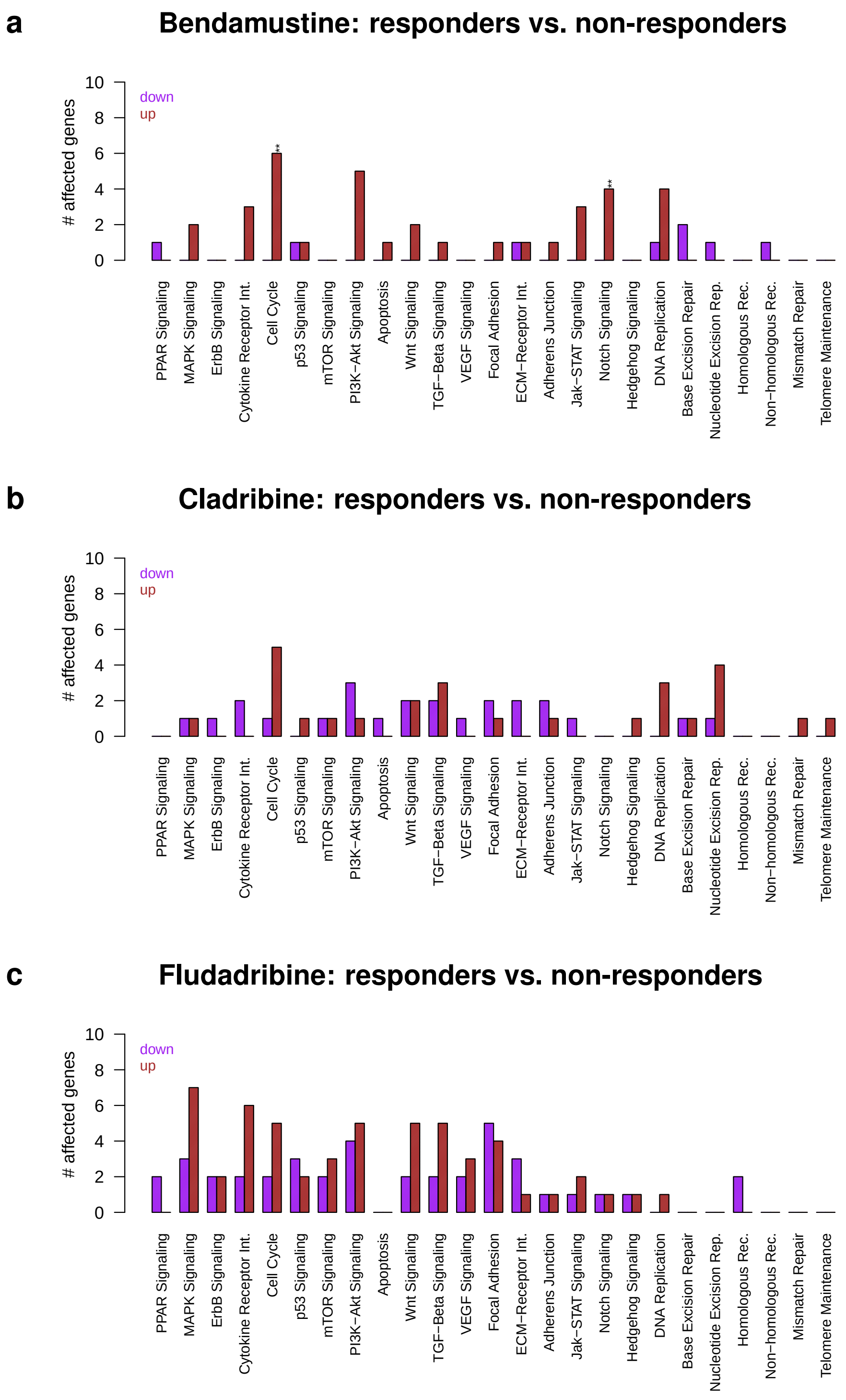

Figure S9: Signaling pathway enrichment analysis of differentially expressed genes between responders and non-responders to three drugs bendamustine (a), cladribine (b), and fludarabine (c). For each drug all down-regulated (lilac) and up-regulated (brown) genes between drug-specific responders and non-responders at the p-value cutoff of 0.05 were considered (Table S3) and analyzed for their membership in known cancer-signaling pathways. The enrichment of differentially expressed genes in a specific pathway was analyzed by Fisher's exact test. Significantly enriched pathways are labeled by asterisks '**' (FDR-adjusted $p \leq 0.01$).
