# Supplementary material for: Comparative preclinical drug response analyses of T-prolymphocytic leukemia reveal no differences between known gene expression subgroups
Source: Biol Direct. 2025 Oct 27;20:106. doi: 10.1186/s13062-025-00701-3 (PMC12557856; doi:10.1186/s13062-025-00701-3)
Supplement: Supplementary file 10 — Supplementary Material 10 [file 13062_2025_701_MOESM10_ESM.pdf]

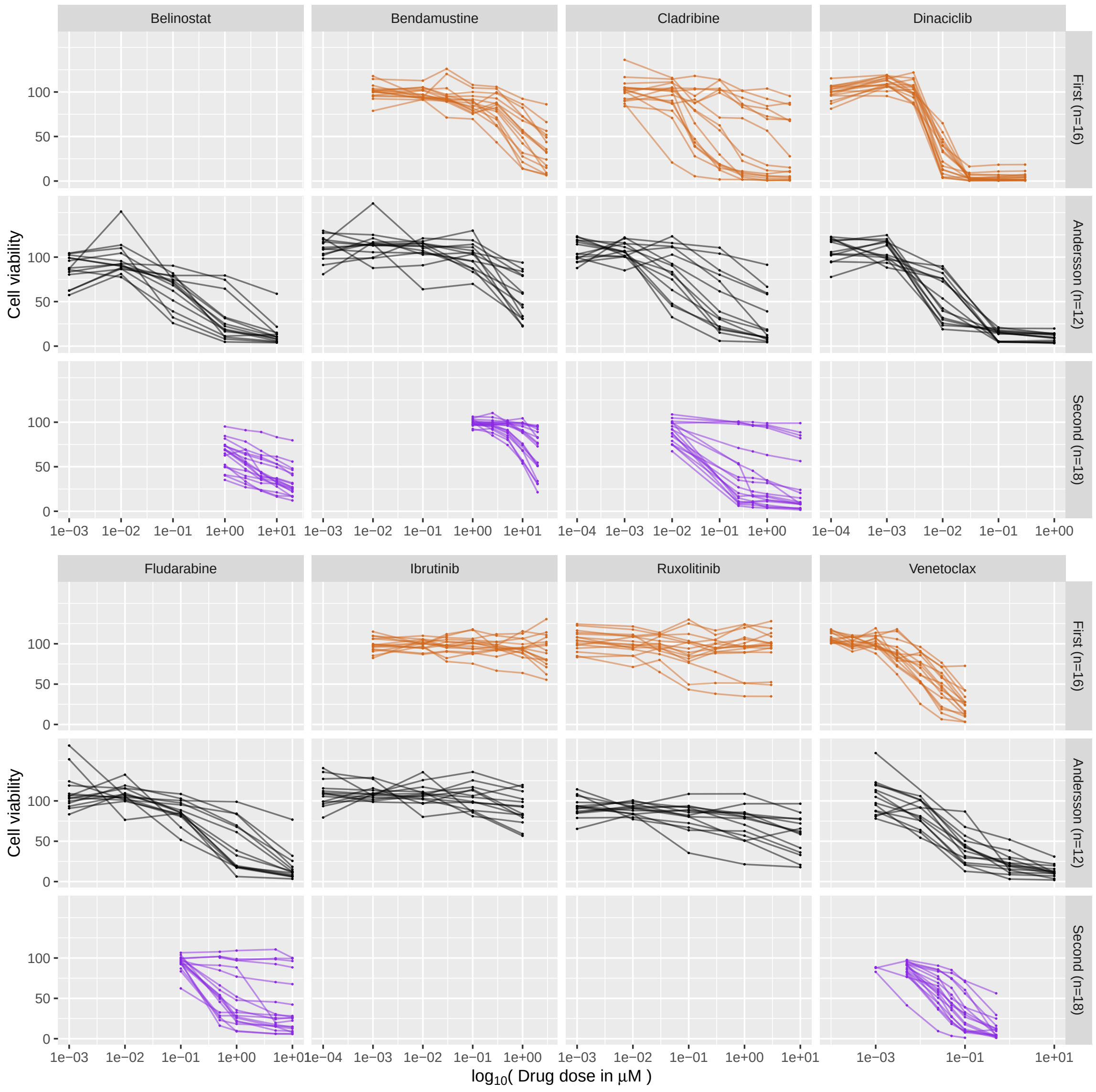

Figure S10: Drug response profiles measured for the three T-PLL cohorts. Cultured peripheral blood mononuclear cells of T-PLL patients were treated with different drugs at different drug doses (x-axis). Cell viabilities were quantified as percentage in relation to the negative control of untreated cells (y-axis). Each curve within a shown subpanel represents the drug response profile of a T-PLL patient to the corresponding drug for a specific T-PLL cohort (brown: first T-PLL cohort, lilac: second T-PLL cohort, black: Andersson T-PLL cohort).
