# Supplementary material for: Comparative preclinical drug response analyses of T-prolymphocytic leukemia reveal no differences between known gene expression subgroups
Source: Biol Direct. 2025 Oct 27;20:106. doi: 10.1186/s13062-025-00701-3 (PMC12557856; doi:10.1186/s13062-025-00701-3)
Supplement: Supplementary file 11 — Supplementary Material 11 [file 13062_2025_701_MOESM11_ESM.pdf]

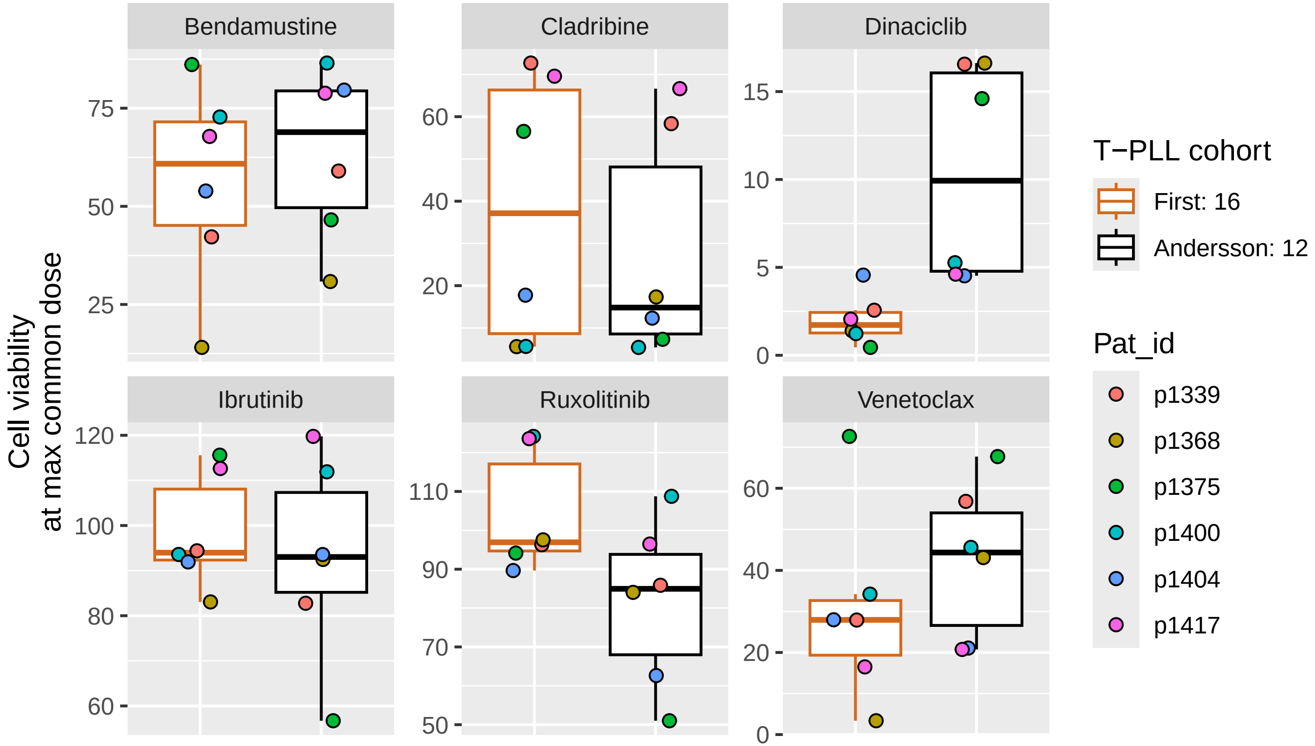

Figure S11: Comparison of the drug response behavior of the overlapping patients of the first T-PLL cohort and the Andersson cohort. Box plots show the cell viabilities of the cultured peripheral blood mononuclear cells of the T-PLL patients measured at the maximal common drug dose of both cohorts (Figure S1). Dots with same color in a specific subpanel of a drug represent the measured cell viabilities for the samples from the same patient in the two cohorts. Significant differences in median cell viabilities of both cohorts were only observed for dinaciclib ($p = 0.03125$) considering a Wilcoxon signed-rank test to account for the paired measurements.
