# Supplementary material for: Comparative preclinical drug response analyses of T-prolymphocytic leukemia reveal no differences between known gene expression subgroups
Source: Biol Direct. 2025 Oct 27;20:106. doi: 10.1186/s13062-025-00701-3 (PMC12557856; doi:10.1186/s13062-025-00701-3)
Supplement: Supplementary file 12 — Supplementary Material 12 [file 13062_2025_701_MOESM12_ESM.pdf]

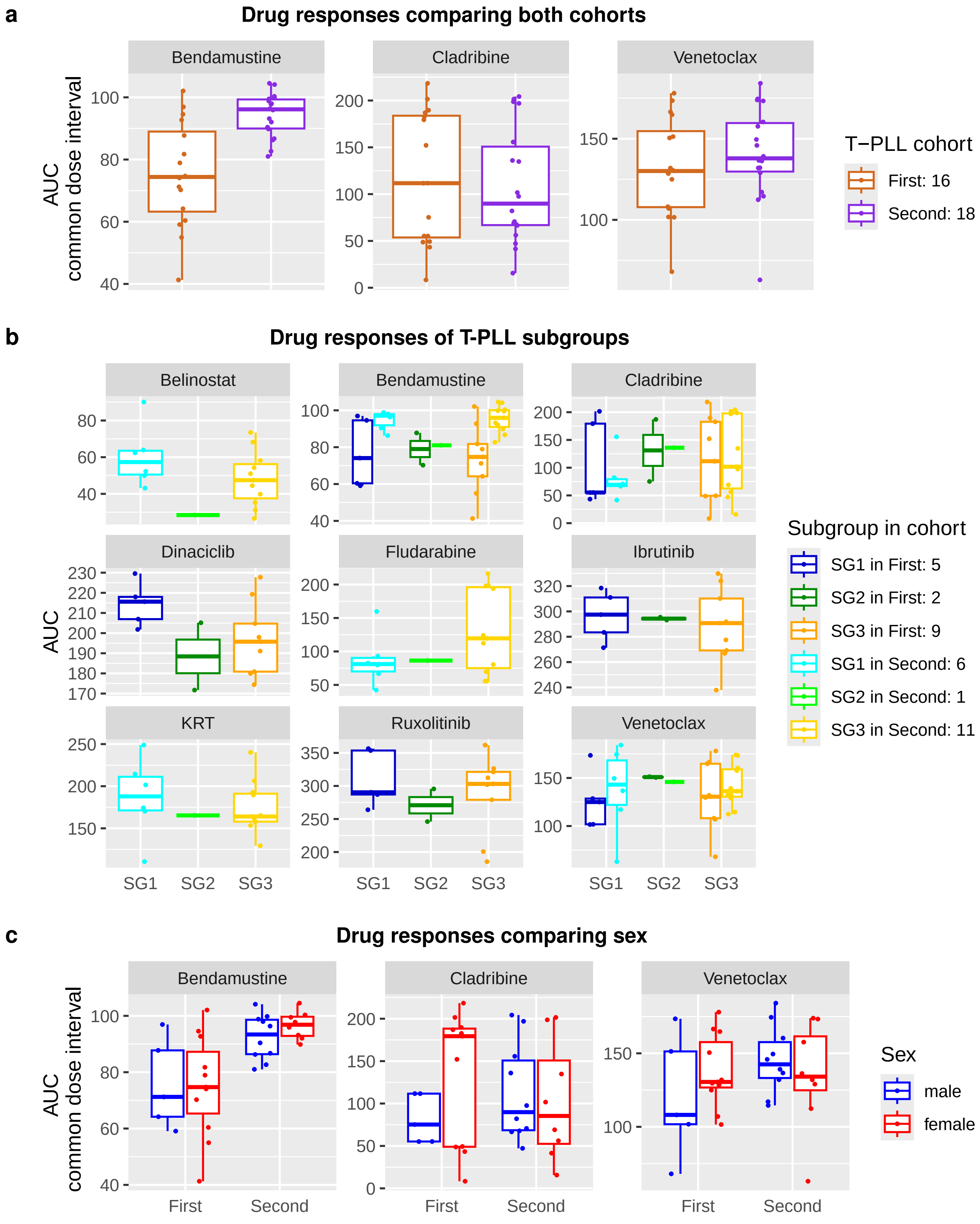

Figure S12: Comparison of drug response behavior of both considered T-PLL cohorts. a, Box plots of the area under the drug response curve AUC obtained for the longest common dose interval of a specific drug tested in both cohorts. b, Box plots of AUC values stratified according to the assignments of patients to known T-PLL gene expression subgroups. For the three drugs bendamustine, cladribine, and venetoclax tested in both cohorts, AUCs obtained for the longest common dose interval of both cohorts are shown and for the other six drugs that were only tested in one cohort AUCs obtained for the whole drug dose interval are shown. c, Box plots of AUCs obtained for the longest common dose interval of both cohorts stratified according to sex of patients.
