# Supplementary material for: Comparative preclinical drug response analyses of T-prolymphocytic leukemia reveal no differences between known gene expression subgroups
Source: Biol Direct. 2025 Oct 27;20:106. doi: 10.1186/s13062-025-00701-3 (PMC12557856; doi:10.1186/s13062-025-00701-3)
Supplement: Supplementary file 13 — Supplementary Material 13 [file 13062_2025_701_MOESM13_ESM.pdf]

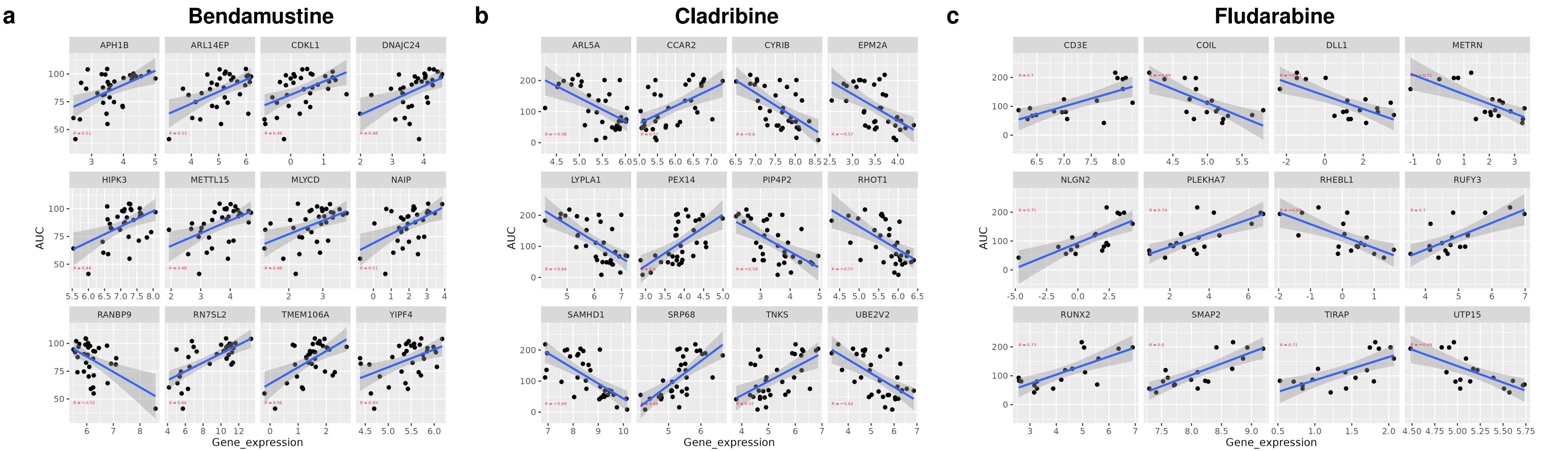


**Figure S13:** Scatterplots between gene expression levels and patient-specific drug responses of the top-12-ranking genes of each drug. For bendamustine and cladribine tested in both cohorts, AUCs obtained for the longest common dose interval of both cohorts are shown and for fludarabine that was only tested in one cohort AUCs obtained for the whole drug dose interval are considered.
