# Supplementary material for: Comparative preclinical drug response analyses of T-prolymphocytic leukemia reveal no differences between known gene expression subgroups
Source: Biol Direct. 2025 Oct 27;20:106. doi: 10.1186/s13062-025-00701-3 (PMC12557856; doi:10.1186/s13062-025-00701-3)
Supplement: Supplementary file 14 — Supplementary Material 14 [file 13062_2025_701_MOESM14_ESM.pdf]

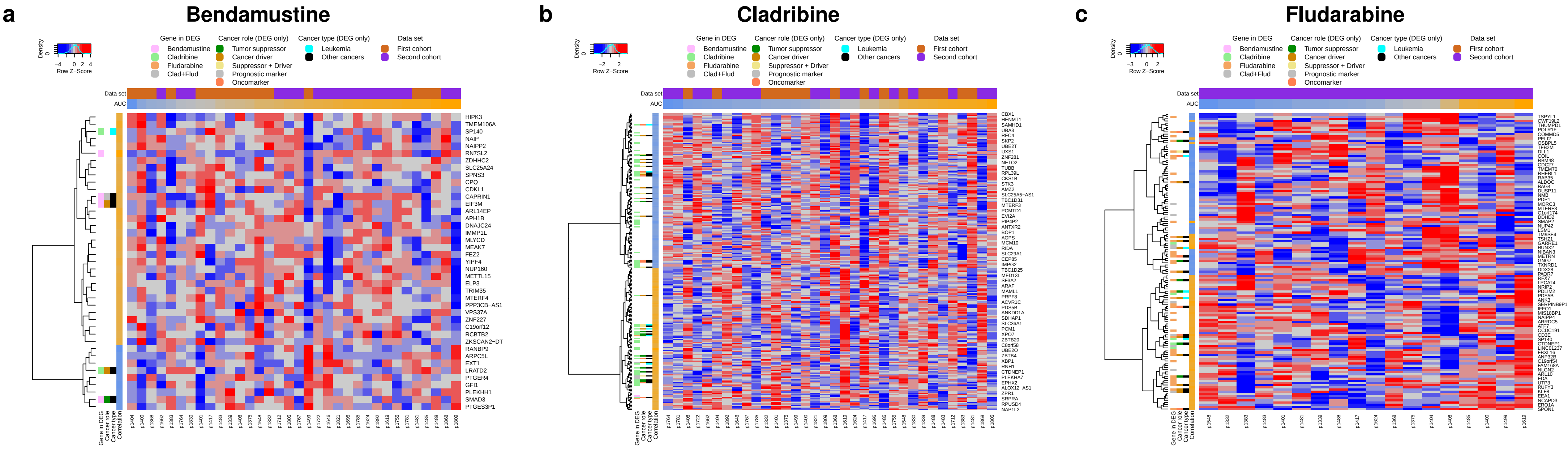

Figure S14: Heatmaps of gene expression levels of top-ranking candidate genes correlated with drug responses. All top-ranking genes up to a p-value cutoff of 0.01 (Table S8) are included for each of the three drugs. The gene expression data were obtained from the T-PLL patient samples prior to treatment independent of the drug response analysis. The individual cells of the heatmap represent z-scores of the $\log_2$-expression values of the genes across the patient samples scaled per row. The columns represent the T-PLL patients in ascending order of their specific drug response quantified by AUC values. The data set bar above the heatmap shows if a T-PLL patient was part of the first or the second T-PLL cohort. The rows of the heatmap that represent the genes were hierarchically clustered. Color coding bars on the left side of the heatmap highlight gene functions and known roles in cancer for genes that overlap with the genes predicted by the differential gene expression analysis comparing potential responders and non-responders. No additional in-depth annotation analysis was done for the other genes that did not overlap with the differential gene expression analysis. In subpanels b and c only some names of the included genes are shown due to space limitations. See Table S8 for all gene names. Obtained correlations between gene expression levels and AUC values across all patients are illustrated as color coding bar left to each heatmap with colors from blue to orange for increasing correlation values. The same color scheme was used to represent the patient-specific AUC values shown as color coding bar above each heatmap.
