# Supplementary material for: Comparative preclinical drug response analyses of T-prolymphocytic leukemia reveal no differences between known gene expression subgroups
Source: Biol Direct. 2025 Oct 27;20:106. doi: 10.1186/s13062-025-00701-3 (PMC12557856; doi:10.1186/s13062-025-00701-3)
Supplement: Supplementary file 23 — Supplementary Material 23 [file 13062_2025_701_MOESM23_ESM.docx]

• Supplementary Table S1: Details to the tested drugs. (.xls)

• Supplementary Table S2: Drug response profiles of tested drug doses for both

considered T-PLL cohorts and the additional validation cohort. (.xlsx)

• Supplementary Table S3: Drug-specific differential gene expression analysis

comparing responders to non-responders. (.xlsx)

• Supplementary Table S4: Gene annotation and literature analysis results of

differentially expressed genes. (.xlsx)

• Supplementary Table S5: Gene expression data of the T-PLL patients of both

T-PLL cohorts. (.xlsx)

• Supplementary Table S6: Pathway annotations of genes utilized for functional

enrichment analyses. (.xlsx)

• Supplementary Table S7: Patient-specific values of the area under the drug

response curve for the longest common dose interval shared between the cohorts in

which a drug was tested. (.xls)

• Supplementary Table S8: Gene-specific correlations between gene expression

levels and drug responses for bendamustine, cladribine, and fludarabine. (.xlsx)
